# From Leuco to Blue: Photochemical Redox Amplification for Small-Molecule Immunodetection

**DOI:** 10.1021/jacs.6c06373

**Published:** 2026-06-08

**Authors:** Tamara Moya-Cavas, Elena Benito-Peña, Arev Sargsyan, Antonio Abad-Fuentes, Guillermo Orellana

**Affiliations:** † Department of Analytical Chemistry, Faculty of Chemistry, Complutense University of Madrid, Plaza Ciencias 2, 28040 Madrid, Spain; ‡ Institute of Agrochemistry and Food Technology, Spanish Council for Scientific Research (IATA-CSIC), Av. Agustí Escardino 7, 46980 Paterna (Valencia), Spain; ⊥ Department of Organic Chemistry, Faculty of Chemistry, Complutense University of Madrid, Plaza Ciencias 2, 28040 Madrid, Spain

## Abstract

Sensitive enough detection of small-molecule toxins and many other target analytes remains challenging for immunoassays that rely on stoichiometric reporters or enzymatic cascades. Here we introduce an enzyme-free, light-gated amplification strategy that teams up stabilized leucomethylene blue (LMB) derivatives with antibody recognition and photoredox cycling. As a proof-of-concept, we designed a stable LMB precursor (δ-BLMB) and a BLMB–zearalenone (ZON) conjugate that undergo rapid, selective photo-oxidation to methylene blue (MB) only under the assay illumination. Electrochemical analysis has established δ-BLMB as a redox precursor to MB, while time-resolved luminescence of [Ru­(phen)_3_]^2+^ (RP3) photocatalyst revealed diffusion-limited quenching (*k*_q_ = 2.68 × 10^9^ M^–1^ s^–1^) consistent with dynamic electron transfer to the precursor. Mechanistic studies show that blue light activates RP3 to generate acyl-radical and MB^–^ intermediates via O_2_-independent pathways; red light is then used to excite nascent MB to drive a secondary photocatalytic reductive cycle that regenerates MB through radical/anionic intermediates, yielding exponential signal growth. We have validated immunoassay integration using ZON-specific monoclonal antibodies in bead-based and plate formats, demonstrating specific, light-triggered fluorescence that reports competitive binding. Its implementation with sequential two-color illumination demonstrates selective system activation and precise control of the signal amplification (300-fold). This photochemical approach provides stable, long-term reagents that can be tailored to the target species and offers a potentially general strategy for developing enzyme-free, high sensitivity immunoassays for small analytes.

## Graphical abstract

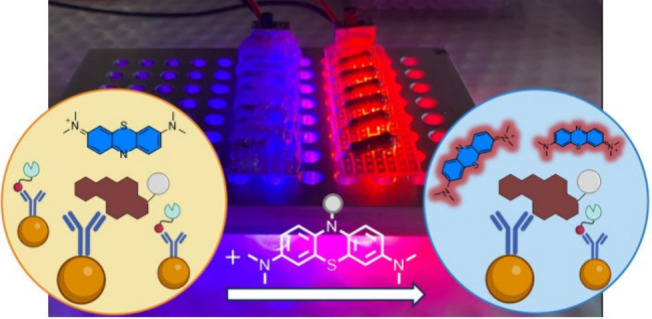

Detection of trace molecules has driven analytical chemistry to develop amplification strategies that translate weak recognition events into sizable optical or redox signals. Among these, photochemical amplification is particularly attractive because it directly converts photonic energy into measurable outputs, often via catalytic feedback cycles. Over the past decade, photoinduced mechanisms—namely photochromic switching, singlet dioxygen-mediated machinery, and redox photocatalysis—have markedly improved sensitivity for protein and pathogen assays.^1,2^ For example, some systems use boron-dipyrromethene (BODIPY) dyes that are revived by reactive oxygen species, continuously regenerating the photosensitizer and amplifying singlet dioxygen production.^3,4^ Other schemes rely on dyes such as eosin Y, regenerated by photoinduced electron transfer, to sustain autocatalytic cycles that push signals beyond the stoichiometric limits.^3^

In this context, attractive photocatalysts should display efficient visible-light absorption, long-lived (triplet) excited states, and the ability to support autocatalytic redox cycles.^5^ Their performance can be evaluated with classical tools such as Stern–Volmer analysis and photon-to-signal gain studies.^6^ Among them, the methylene blue (MB)/leucomethylene blue (LMB) redox pair is especially useful and can be photochemically toggled. MB absorbs strongly in the red to form a reactive triplet state capable of participating in both oxidative and reductive cycles.^6^ Upon reduction to LMB, it can be rapidly reoxidized to MB, enabling regeneration and signal growth.^7,8^ Owing to this reversible chemistry, the MB/LMB pair has been successfully applied to optical sensors, oxygen probes, and bioimaging.^9–12^

However, extending the MB/LMB cycle to boost sensitivity of immunoassays for toxin or other trace analytes quantification is not trivial. Leuco derivatives are typically unstable to air, undergoing spontaneous oxidation that leads to signal drift and complicates storage.^13^ Moreover, direct conjugation of MB or LMB to small haptens might interfere with antibody recognition and destabilize the controlled redox behavior required for reproducible photoamplification. To address these challenges, we designed and prepared *N*-acylated LMB derivatives that are stable under ambient conditions yet undergo rapid, selective oxidation to MB. These derivatives might yield high-contrast, fluorescence microscopy readouts with low background,^14^ but we have used them instead to carry out an enzyme-free, photocatalytic redox amplification that multiplies the sensitivity of bioassays and biosensors built on antibody recognition.

As a case in point, we chose zearalenone (ZON), a widespread foodborne mycotoxin responsible for estrogenic and reproductive problems.^15–17^ We prepared *N*-acylated LMB conjugates and integrated them into two architectures: (i) LMB linked to the hapten for competitive assays, and (ii) LMB attached to a secondary antibody for indirect assay formats. We evaluated their chemical stability, redox behavior, and photochemical amplification features relative to the non-enhanced runs. Although this initial study is focused on ZON, its principles are transferable to other small analytes that can be functionalized without blocking their antibody-recognition sites. In this way, we started by synthesizing the heretofore unknown *N*-chloromethylbenzoyl leucomethylene blue (δ-BLMB), an O_2_- and light-stable derivative that allows its further conjugation to ZON (BLMB–ZON) (Figure 1).

**Figure 1:**
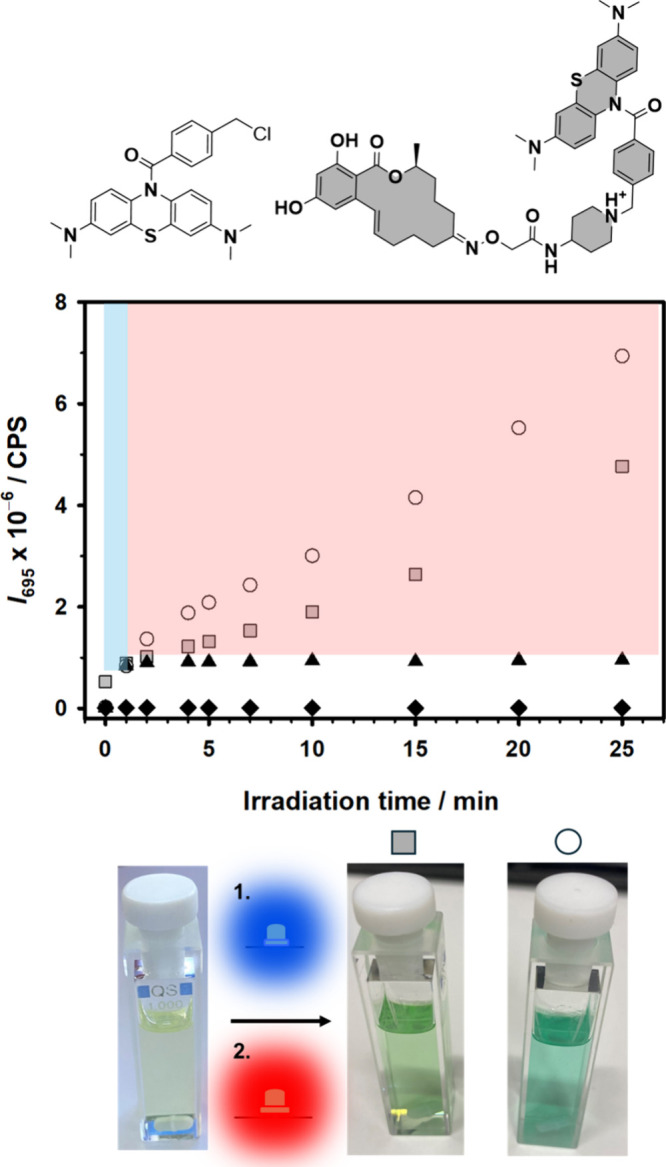
Structures of δ-BLMB and of the BLMB-ZON conjugate. Fluorescence intensity at 695 nm (λ_exc_ = 650 nm) after 1 min of 450 nm blue-laser illumination (1.) of 10 μmol L^–1^ RP3 in the presence of δ-BLMB (50 μmol L^–1^, ◯) or the BLMB-ZON conjugate (50 μmol L^–1^, ■), as a function of the additional irradiation time with a 650 nm red diode laser (2.). The initial yellow solutions in aerated ACN (bottom left), due to the RP3 complex, turn blue-green (bottom right) showing the methylene blue formation. Control experiments with RP3 and δ-BLMB after 1 min irradiation with a 450 nm blue laser followed by monitoring in the dark (▲), and with just the 650 nm red laser from *t* = 0 (◆), are shown as well.

The electrochemical characterization of δ-BLMB and related species (Figures S17 and S18) provided key insights into their redox properties. Cyclic voltammetry delineated oxidation–reduction windows and confirmed that δ-BLMB is electrochemically converted back to MB at potentials >0.6 V. The sharp peak at ca. 0.72 V (Figure S18C) just indicates adsorption phenomena or subsequent surface reactions.^18,19^ A comparison with the MB voltammogram (Figure S17B) corroborated that the peaks observed after the first reduction correspond to the MB formation, demonstrating that δ-BLMB serves as an appropriate redox precursor of MB.

Analysis using different applied potential windows (Figure S18) clarified the electrochemical sequence and enabled assignment of the redox couples involved (δ-BLMB/MB^+^/MB^• 0^/MB^–^). From the corresponding experimental half-wave potentials (*E*_0_) and the luminescence maximums (λ_max_) (Table S1), we have calculated the excited-state redox potentials using the equation *E = *E*_0_ ± (*hc*/q_e_λ_max_), where *h*, *c* and q_e_ are the Planck constant, the speed of light and the electron charge, respectively. We can observe that all the suggested redox processes are thermodynamically feasible, as they show Δ*E* > 0 (Figure S19).

The initial quenching of the [Ru­(phen)_3_]^2+^ (RP3) photocatalyst by δ-BLMB (Figure S19) has been evidenced from luminescence lifetime measurements in the absence (τ_0_) and in the presence (τ) of the quencher (Figure S20). Progressive additions of δ-BLMB significantly decreased the excited-state lifetime of RP3, yielding a linear Stern–Volmer relationship (τ_0_/τ = 1 + *k*_q_τ_0_[Q]) with *k*_q_ = 2.68 × 10^9^ L mol^–1^ s^–1^, a rate constant consistent with near diffusion-controlled photoinduced electron transfer.^20^ Moreover, after extended illumination with a blue laser diode, the RP3 lifetime partially recovered, in agreement with the photochemical formation of MB from δ-BLMB (reactions II and III, Figure S19).

With the electrochemical and photophysical basis established, we evaluated solution-phase photochemical amplification (Figure 1). Taking into account the efficient photogeneration of singlet dioxygen by ruthenium­(II) polypyridyls,^21^ we irradiated δ-BLMB solutions with and without O_2_ using RP3 (Figure S21A) to test the involvement of reactive oxygen species (ROS) in the δ-BLMB to MB conversion. Interestingly, MB formation proceeded equally or somewhat more efficiently under oxygen-free conditions (Figure S21B), indicating a ROS-independent mechanism compatible with direct photoinduced electron transfer. Control experiments lent support to this fact: using MB as the sole photocatalyst, red illumination did not produce additional MB from δ-BLMB (Figure S21C), demonstrating that redox potential differences prevent self-regeneration with this composition. Furthermore, red-light illumination of the RP3/δ-BLMB mixture (Figure 1) did not lead to any amplification either. Therefore, amplification requires a specific photocatalyst (RP3 or another complex with a similar excited state redox potential) to initiate oxidation of the leuco derivative; for instance, despite the quantitatively generated long-lived triplet of a palladium porphyrin, no photocatalytic amplification is observed either in the absence or in the presence of O_2_ (Figure S21D). Figure S21C also demonstrates that no photodegradation of MB occurs during the red illumination time in aerated acetonitrile.

After considering the above-mentioned facts and the experimental redox potentials (Table S1), the photocatalytic mechanism for the amplification system shown in Figure 2 is proposed. According to this mechanism, photoamplification requires a sequential two-color illumination: a short blue-light irradiation to excite RP3 and generate a small amount of MB (MB^+^), followed by red-light irradiation that selectively excites MB to self-generate this species. In this way, the MB fluorescence at 695 nm steadily increases from δ-BLMB (and from the BLMB–ZON conjugate as well) (Figure 1); visually, yellow solutions turn blue green, consistent with MB accumulation. The absorption spectra of the solutions after illumination (Figure 3) showed new bands in the 600–700 nm region, characteristic of MB. Nevertheless, δ-BLMB yielded a stronger color change and fluorescence enhancement than the ZON conjugate for the same illumination time. This fact may be attributed to the phenolic groups of the latter that behave as radical scavengers, slowing down the redox cycle.^22,23^

**Figure 2:**
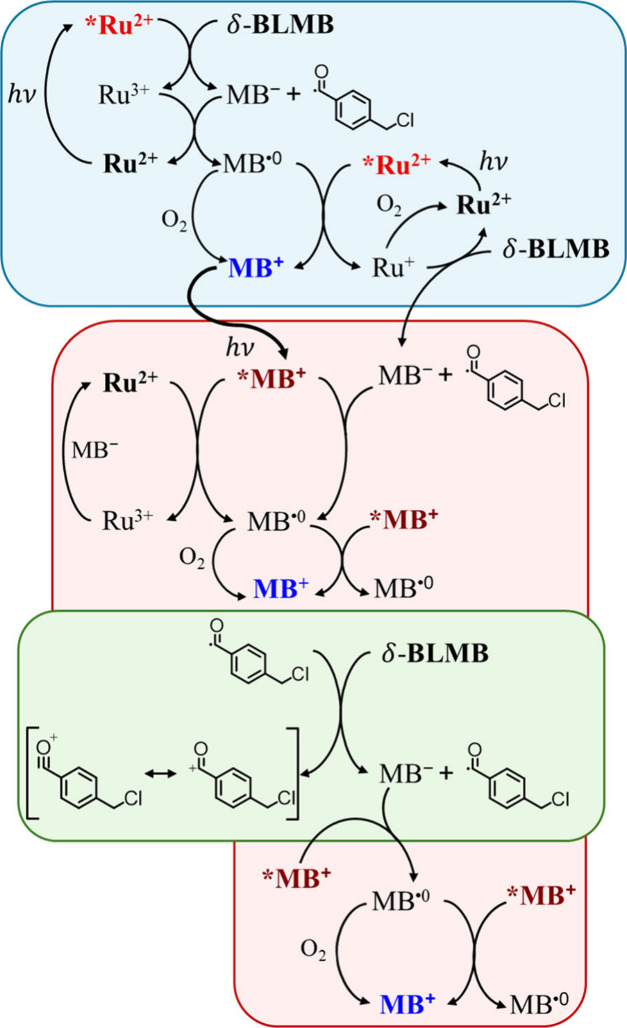
Proposed redox photocatalytic mechanism for the amplification system. Reactions are grouped in colored boxes according to the conditions under which they occur: blue boxes indicate reactions induced by blue light; reactions into red boxes occur upon red light illumination, and reactions within green boxes correspond to those occurring after the addition of the pro-photocatalyst. Species highlighted in red color appear only under blue light illumination, while the species in brown color are those activated by red light. The blue color highlights methylene blue (MB), the fluorescence of which is monitored as the analytical signal, confirming redox photocatalytic amplification. Added species (photocatalyst and pro-photocatalyst) are shown in black bold typeface.

**Figure 3:**
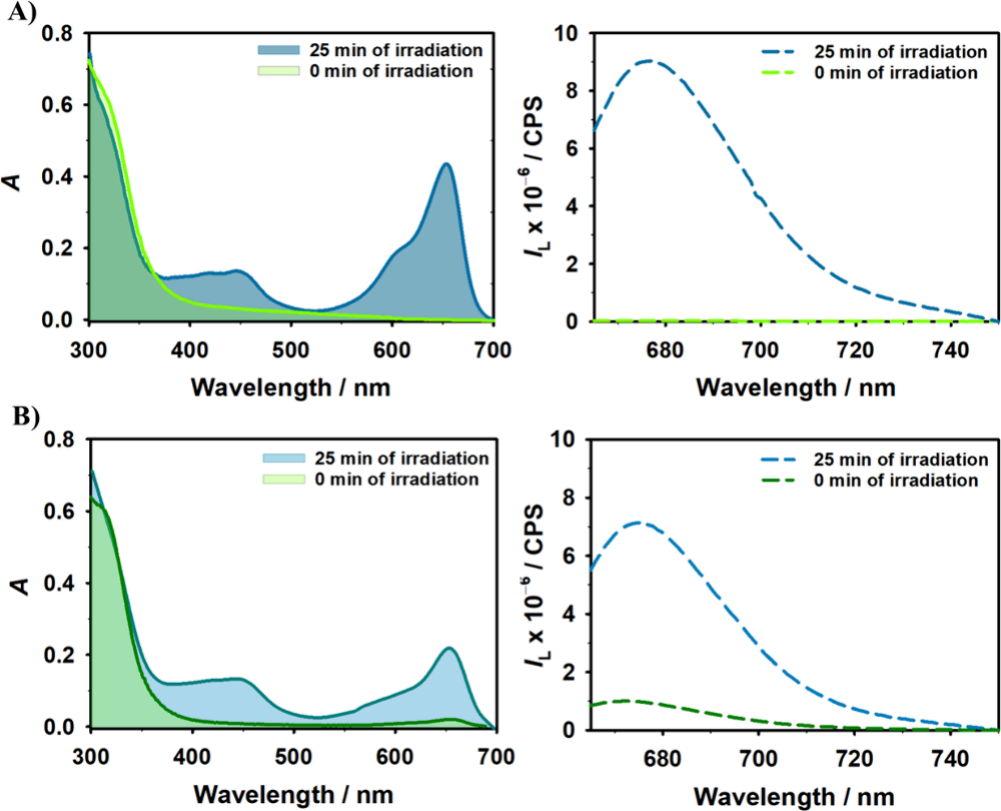
Absorption (left) and emission (λ_exc_ = 650 nm; right) spectra before (green lines) and after (blue lines) 1 min of 450 nm blue-laser illumination followed by 24 min of 650 nm red-laser irradiation of 50 μmol L^–1^ δ-BLMB (A) or 50 μmol L^–1^ BLMB-ZON conjugate (B), both in the presence of 10 μmol L^–1^ RP3 in ACN. The ZON conjugate exhibited residual MB absorption/emission before illumination, due to some oxidation during its synthesis.

The proposed MB^• 0^ reoxidation pathways (Figure 2) involve the dissolved O_2_ but may also occur in the absence of it by alternative routes. This is why photoamplification takes place even in deoxygenated solution (Figure S21B). The fact that a slightly higher amplification occurs under O_2_-free conditions is probably due to the longer lifetime of *Ru­(phen)_3_^2+^, because it is a consequence of the blue illumination step. However, amplification is suppressed in water possibly due to protonation of MB^–^ (p*K*_a_ = 5.8)^24^ or reaction of the acyl radicals with water.^25^

For a practical assay amplification protocol, we built a blue/red LED illuminator (Figure S1) for 96-well white plates (Figure S22). For the sake of the experiment time, a red diode laser rather than an LED was used for amplification. A similar swap was not needed for the blue illumination of the RP3 photocatalyst.

As summarized in Figure 4, the proof-of-concept photoamplified immunoassay couples molecular recognition with light-driven redox cycling. Initial competition occurs between free ZON and the BLMB-ZON conjugate for the antibody binding sites. After solvent exchange from aqueous PBS buffer to ACN, the photocatalyst (RP3) is added which, upon blue LED irradiation, reduces the BLMB-ZON conjugate bound to the (Ab#4) antibody to MB^–^. Then, some δ-BLMB (“pro-photocatalyst”) is introduced and, under red-light illumination, the very small amount of methylene blue generated during the initial photocatalytic step is excited and acts as a secondary photocatalyst. This process generates both additional acyl radicals, which reduce δ-BLMB and generate methylene blue (Figure 2), thereby amplifying the analytical signal of the assay. For instance, under the experimental conditions indicated in Figure 4, the photoamplification factor amounts to ca. 300-fold but, under proper optimization, it might reach 3 orders of magnitude. The versatility of the proposed amplification mechanism was also demonstrated in a competitive inhibition immunoassay for ZON using instead a BLMB-labeled rabbit antimouse antibody (Figure S25).

**Figure 4:**
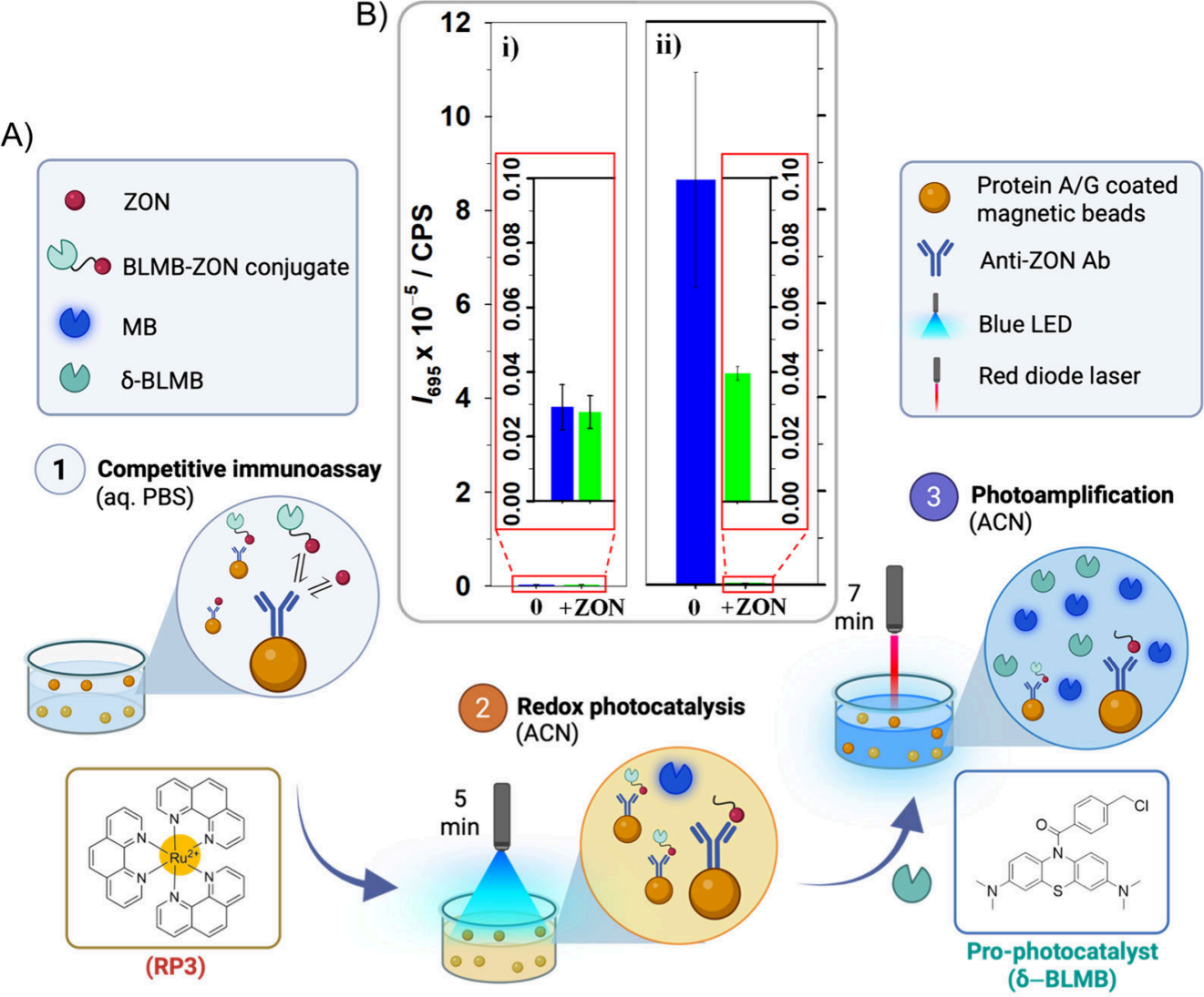
(A) Scheme of the photoamplified assay in heterogeneous medium (magnetic beads/microplate). (B) Fluorescence of MB at 695 nm using 1 μmol L^–1^ BLMB-ZON conjugate, in the presence (+ZON) and in the absence (0) of 10 μmol L^–1^ ZON, according to the assay described in panel (A) (*n* = 3); (i) immediately after 5 min of blue-LED irradiation and addition of 153 μmol L^–1^ δ-BLMB (pro-photocatalyst); (ii) after subsequent red-diode laser illumination for 7 min.

While further optimization is needed, particularly of the bioassay media and light delivery, our initial results validate replacing enzymatic cascades with photoredox amplification. The integrated synthesis, electrochemical analysis, and photophysical characterization provide a solid foundation for next-generation immunoassays targeting small molecules such as foodborne mycotoxins, with improved sensitivity and a tunable dynamic range. Future work will seek to perfect the assay components, concentration and illumination times, integrate microfluidics, and extend the photoamplification to other trace-level target haptens.

## Supplementary Material



## Notes

Experimental details, synthetic procedures, chemicals characterization data, spectroscopic and mass spectrometric analyses, electrochemical and photocatalytic studies, mechanistic analysis, bioassay and amplification assay results, including Figures S1–S25 and Table S1 (PDF)
