# Supplementary material for: From Leuco to Blue: Photochemical Redox Amplification for Small-Molecule Immunodetection
Source: J Am Chem Soc. 2026 Jun 8;148(23):23469–73. doi: 10.1021/jacs.6c06373 (PMC13281526; doi:10.1021/jacs.6c06373)
Supplement: Supplementary file 1 [file ja6c06373_si_001.pdf]

Supplementary Information

From Leuco to Blue: Photochemical Redox Amplification for Small-Molecule Immunodetection

Tamara Moya-Cavas,[†] *Elena Benito-Peña,*[†] *Arev Sargsyan,*[‡] *Antonio Abad-Fuentes,*[‡] *Guillermo Orellana**[,⊥]

[†] Department of Analytical Chemistry, Faculty of Chemistry, Complutense University of Madrid, Plaza Ciencias 2, 28040 Madrid, Spain

[‡] Institute of Agrochemistry and Food Technology, Spanish Council for Scientific Research (IATA-CSIC), Av. Agustí Escardino 7, 46980 Paterna (Valencia), Spain.

[⊥] Department of Organic Chemistry, Faculty of Chemistry, Complutense University of Madrid, Plaza Ciencias 2, 28040 Madrid, Spain;

Email: orellana@quim.ucm.es

EXPERIMENTAL

Reagents

Various reagents and solvents were used in the syntheses and experiments described in this section. HPLC-grade solvents, including dichloromethane (DCM), chloroform, ethyl acetate, methanol (MeOH), ethanol (EtOH), dimethylformamide (DMF), acetonitrile (ACN), diethyl ether and cyclohexane, were purchased from Fisher Scientific (Waltham, MA). Purified water was obtained from a Merck-Millipore Direct Q-3UV laboratory system (Bedford, MA), while deuterated solvents were purchased from Avantor (Radnor, PA). Analytical grade trifluoroacetic acid (TFA, 99%) was supplied by Fluorochem (Glossop, UK), while hydrochloric acid (37%) was from Fisher. Anhydrous disodium and monosodium phosphates (Fisher), tris(1,10-phenanthroline)ruthenium(II) dichloride hexahydrate (98%, Merck, Darmstadt, Germany) and [*meso*-tetra(pentafluorophenyl)porphyrin]palladium(II) (PdTFPP) (Frontier Scientific, Logan, UT) were used as received.

The following reactants were obtained from the indicated vendors to prepare the target molecules: Merck supplied methylene blue trihydrate (80% dye content, as determined by ^{1}H-NMR), *N,N*-diisopropylethylamine (100%), and *p*-methoxyphenol (hydroquinone monomethyl ether). Scharlau (Barcelona, Spain) provided sodium carbonate (96%), anhydrous sodium sulphate (99%), and silica gel (0.04–0.06 mm for flash column chromatography). TCI supplied 4-(chloromethyl)benzoyl chloride (99%), 4-(*N*-Boc-amino)piperidine (99%), zearalenone (ZON) (97%), pyrrolidine (100%), and *o*-(carboxymethyl)hydroxylamine hemichloride (98%). Avantor supplied sodium dithionite (85%), while Alfa Aesar (Ward Hill, Massachusetts, USA) provided 1,8-bis(dimethylamino)naphthalene (98%) and [dimethylamino-(3-oxo-1,2,3-triazolo[4,5-b]pyridin-3-ium-1-yl)methylidene]dimethylazanium hexafluorophosphate (HATU, 99%). Protein A/G-functionalized and CO_2H-functionalized magnetic particles were from Cytiva (Marlborough, MA).

The following items were used for the immunoassays: zearalenone-conjugated ovalbumin (OVA-ZON), horseradish peroxidase (HRP)-conjugated zearalenone (HRP-ZON), and zearalenone-selective immunoglobulin G (IgG) monoclonal antibodies Ab#1, Ab#2, Ab#3, and Ab#4 were produced at the Institute of Agrochemistry and Food Technology (IATA-CSIC);[1] Tween-20

(Merck), bovine serum albumin fraction V (NZYtech, Lisbon, Portugal), HRP-conjugated anti-mouse immunoglobulin (HRP-RAM, AffiniPure Jackson, West Grove, PA), and 1-Step Ultra TMB-ELISA (Thermo Scientific, Waltham, MA). NAP Sephadex G-25 DNA-grade columns for purification from Cytiva were also used, as well as Amicon Ultra ultrafiltration devices from Merck.

Equipment and measurement conditions

UV–vis absorption spectra were recorded on a Varian Cary 3-Bio spectrophotometer (Palo Alto, California). Luminescence spectra were measured using either a FluoroSENS spectrofluorimeter (Gilden Photonics, Helensburgh, UK) or a Horiba Fluoromax-4 TCSPC (Kyoto, Japan). Both instruments were equipped with a red-sensitive R928 photomultiplier (Hamamatsu, Japan) and a 150-W xenon lamp. Electrochemical characterization of the compounds was carried out on a PGZ 402 Dynamic EIS system (VoltaLab, Madrid, Spain). In all cases, measurements were performed in argon-sparged acetonitrile (ACN) containing 0.1 mol L^{-1} tetrabutylammonium hexafluorophosphate ($TBAPF_6$), using a standard three-electrode setup consisting of a 3 mm diameter glassy carbon working electrode (GCE), a platinum wire counter electrode and a silver wire reference electrode. For immunoassays, a BMG Labtech CLARIOstar microplate reader (Ortenberg, Germany) was used in luminescence mode. An excitation wavelength of 650 ± 10 nm was used, with plate shaking at 250 rpm for 5 s before each measurement. Additionally, a NanoDrop One spectrophotometer (Thermo Scientific) was used to measure antibody concentration. Emission spectra were not corrected for the instrument response.

Microwave-assisted syntheses were carried out in a Monowave 200 reactor (Anton Paar, Graz, Austria), using either a 10-mL or a 30-mL reactor. ^{1}H-NMR spectra were recorded using a Bruker DPX 300 MHz BACS-60 or a Bruker AVIII 700 MHz spectrometer (UCM Research Support Centers). Mass spectra were obtained on a Bruker Esquire LC-Ion Trap spectrometer (UCM Research Support Centers). Predicted ^{1}H-NMR spectra were generated with the ACD/Predictor software (v.5.09).

A miniSpin centrifuge (Eppendorf AG, Hamburg, Germany), a HydroFlex plate washer with magnetic support (Tecan, Männedorf, Switzerland), and a 311 Vibromatic vortex mixer (Selecta, Barcelona, Spain) were also used for sample preparation or purification.

For the sake of operation convenience in the photochemical amplification experiments into fluorescence cuvettes, 450-nm (50 mW) and 650-nm (150 mW) lasers (Lilly Electronics, Wuhan, P. R. China and OXLasers, Shanghai, P. R. China, respectively) were used to excite the samples at these wavelengths. For microplate work, six 5-mm blue LEDs (Chanzon, Shenzhen, PR China; 30° viewing angle, 2260–2740 mcd) and six 5-mm red LEDs (Kingbright, Taoyuan, Taiwan; 20° viewing angle, 9400–10200 mcd) were arranged in a row in a custom-built device (Figure S1) (UCM electronics workshop). Alternatively, the red laser was also used for the microplate well illumination.

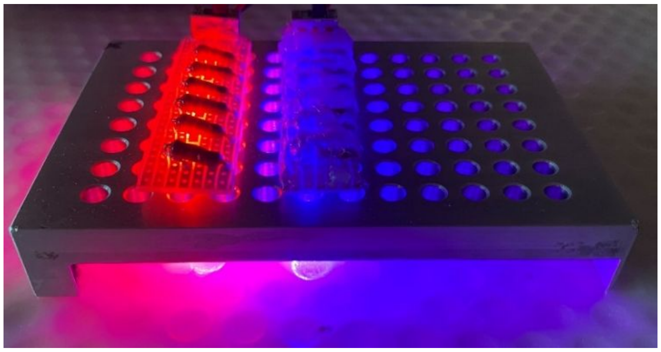

Figure S1. Home-made six-red LED illuminator and six-blue LED illuminator for microplate photoamplification experiments.

Syntheses

Synthesis of the δ-BLMB pro-photocatalyst

The synthesis of δ-BLMB was carried out in three steps (Figure S2). Firstly, 500 mg (1.56 mmol) of MB was dissolved in 14 mL of purified water together with 661 mg (6.24 mmol) of sodium carbonate. Then, 10 mL of DCM were added to this solution under argon and the mixture was heated to 40 °C. Once this temperature was reached, 1.08 g (6.24 mmol) of sodium dithionite, which had been dissolved in 5 mL of water deoxygenated by argon purging, was added under vigorous stirring. After 15 min, the reaction was stopped and the aqueous phase was carefully removed using a syringe fitted with a long needle. The remaining organic phase was dried over anhydrous sodium sulphate.

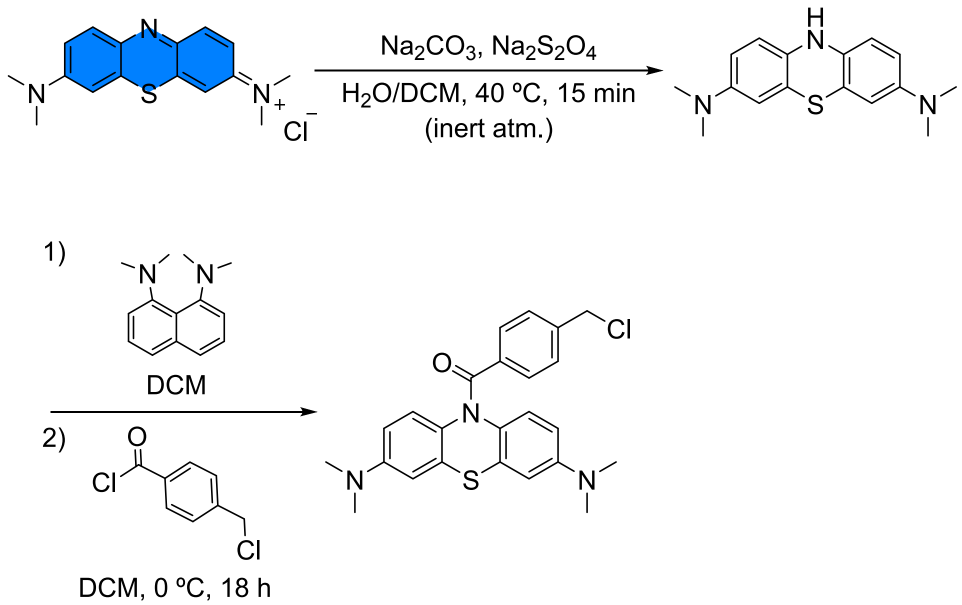

Figure S2. Synthetic route for the introduction of an amino group into δ-BLMB.

Without removing the drying agent, a solution of 1,8-bis(dimethylamino)naphthalene (334 mg, 1.56 mmol) in 2 mL of anhydrous, deoxygenated DCM was added dropwise. After reacting for 15 min into an ice bath, a solution of 4-(chloromethyl)benzoyl chloride (295 mg, 1.56 mmol) in 2 mL of anhydrous DCM was added and the mixture was left to react overnight at room temperature.

To purify δ-BLMB, the reaction mixture was filtered to remove the drying agent and the solvent was evaporated on a rotary evaporator until dry. The resulting raw solid was then washed several times with ethyl acetate to remove the free base. The solid residue was dried using the rotary evaporator and dissolved in the minimum possible volume of chloroform. Then, silica gel was added and the mixture was evaporated under rotary conditions to adsorb the impure product onto the silica. Finally, a 30-mm diameter, 7-cm bed height column was packed using a 1:1 (v/v) hexane–AcOEt, the adsorbed sample was placed on top and the product of interest was eluted with AcOEt. The residual base was retained on the column and a pale green solid was obtained after the eluant evaporation. The product R_f value was 0.78 on silica gel TLC, using a 1:1 hexane–AcOEt eluting system. The purified product was then dried in a vacuum oven at room temperature (0.1 Torr). ^{1}H-NMR (300 MHz, $CDCl_3$): δ/ppm 7.32–7.27 (m, 2H), 7.23–6.99 (m, 6H), 6.91–6.65 (m, 2H), 6.64–6.16 (m, 2H), 4.45 (s, 2H), 2.88 (s, 12H). MS (ESI+) m/z: [M+Na$^+$] calculated for [$C_{24}H_{24}ClN_3OS$ + Na$^+$]:460.12; found: 460.2. The ^{1}H- and ^{13}C-NMR spectra are provided in Figure S3 and the MS spectrum is shown in Figure S4.

Synthesis of the BLMB–ZON conjugate

The BLMB–ZON conjugate was synthesised using a three-step process involving the amination of δ-BLMB, the formation of the zearalenone carboxymethoxyloxime and the amide-type coupling of the two entities. The reaction mixture was maintained at 120 °C for 1.5 h under microwave irradiation with stirring.

i. Amination of δ-BLMB

First, δ-BLMB (100 mg, 0.22 mmol) was dissolved in 10 mL of chloroform together with 4-(*N*-Boc-amino)piperidine (229 mg, 1.15 mmol) in a 30 mL microwave reactor. The reaction mixture was maintained at 120 °C for 1.5 h under microwave irradiation with stirring.

An extraction was then carried out with five portions of water acidified to pH 3 with 1 M HCl, with an additional amount of chloroform being added. To remove the Boc group, 20 mol of TFA per mol of protected amine were added (assuming complete conversion) and the mixture was heated at 60 °C for 2 h (Figure S5). As a result, an emerald-green oil precipitated, which was allowed to cool to promote precipitation of the desired product. The supernatant was then discarded, and the oil washed three times with chloroform. The yield was 100%. The R_f value was 0.58 in 9:1 (v/v) dichloromethane–methanol with two drops of an aqueous ammonia solution. The final product was dried in a vacuum oven at room temperature (0.1 Torr). ^{1}H-NMR (300 MHz, CD_3COCD_3): δ = 7.49 (dd, J_1 = 8 Hz, J_2 = 5.7 Hz, 2H), 7.42–7.36 (m, 2H), 7.22 (d, J = 8.9 Hz, 2H), 6.89 (d, J = 2.8 Hz, 2H), 6.68–6.57 (m, 2H), 3.62 (s, 2H), 3.17 (s, 2H), 2.96 (s, 12H), 2.39 (m, 4H). MS (ESI+) m/z: $[M]^+$ calculated for $[C_{29}H_{35}N_5OS + H^+]$: 502.3; found: 502.3. The ^{1}H-NMR spectrum is provided in Figure S6 while the MS spectrum is shown in Figure S7**.**

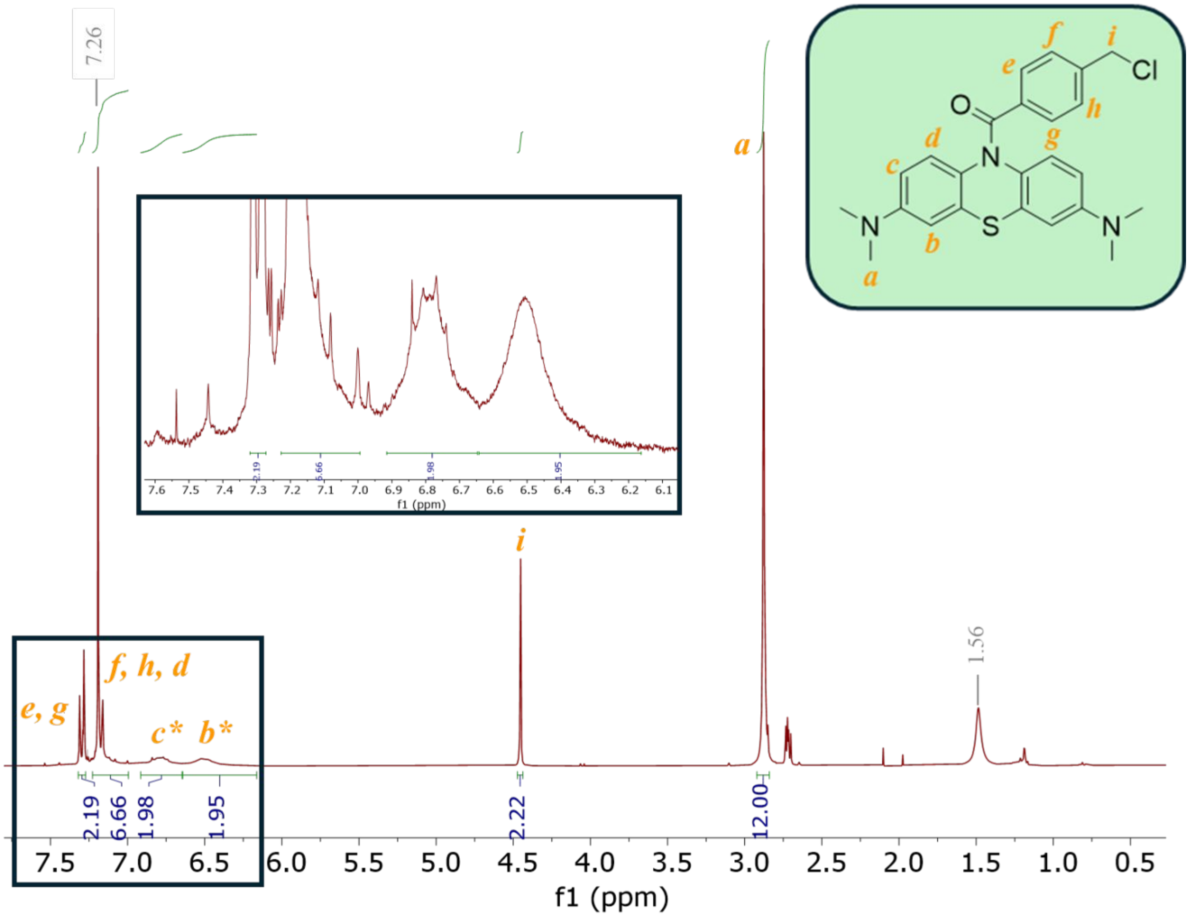

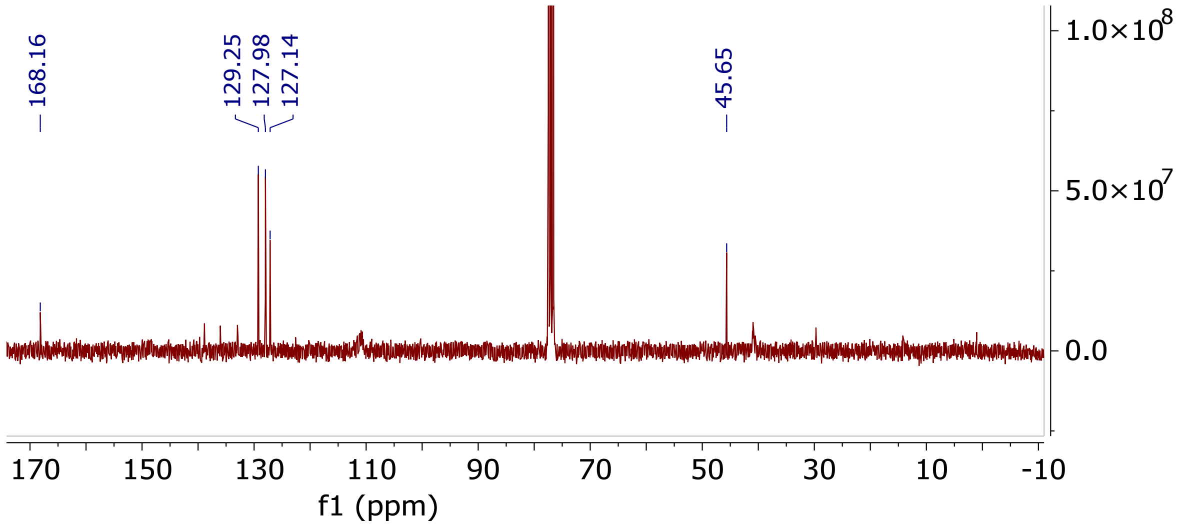

Figure S3. (Top) ^{1}H-NMR spectrum of δ-BLMB recorded in $CDCl_3$ (300 MHz) and (bottom) ^{13}C-NMR spectrum also recorded at 300 MHz in $CDCl_3$. *Tentative assignment.

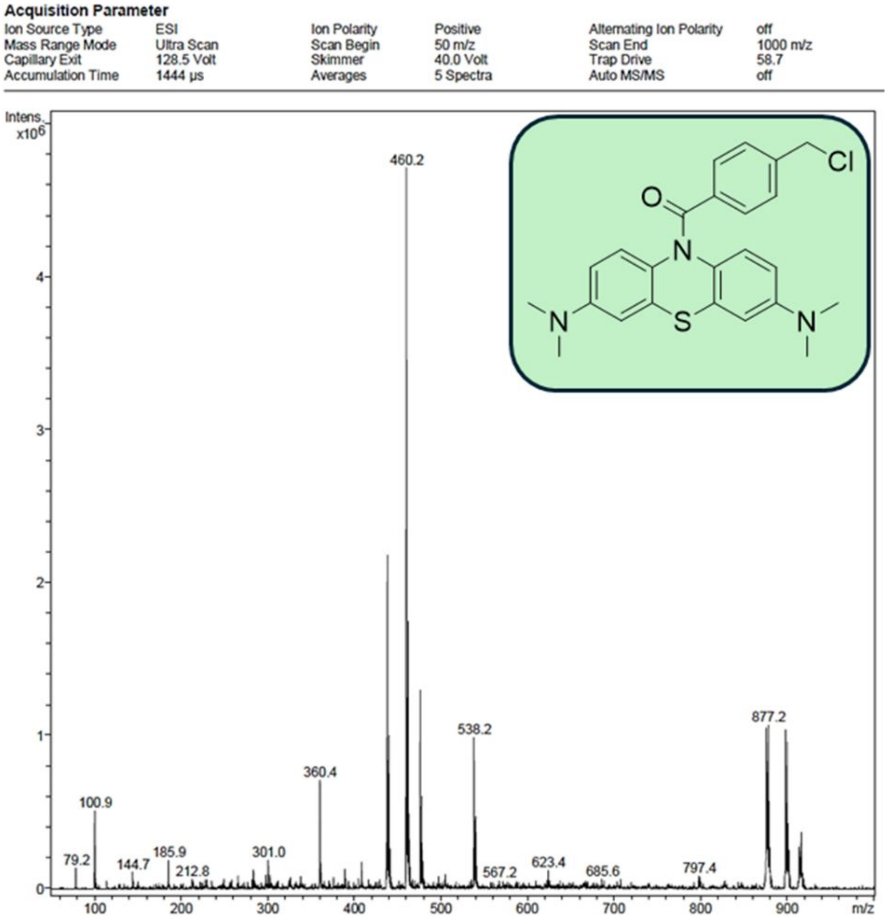

Figure S4. Electrospray ionization mass spectrum (positive ion detection mode) of δ-BLMB, obtained in MeOH; m/z: calculated for [$C_{24}H_{24}ClN_3OS$ + Na]: 460.1; found: 460.2.

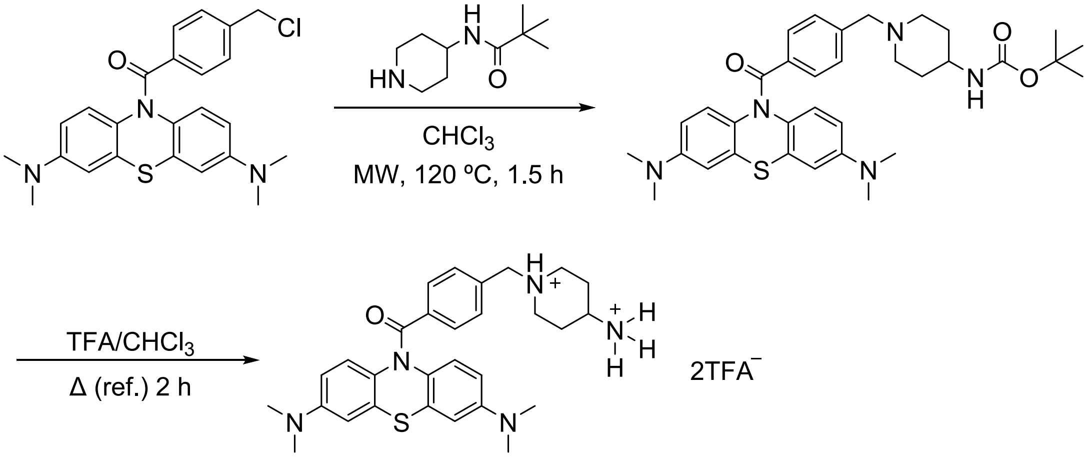

Figure S5. Synthetic route for the introduction of an amino group into δ-BLMB.

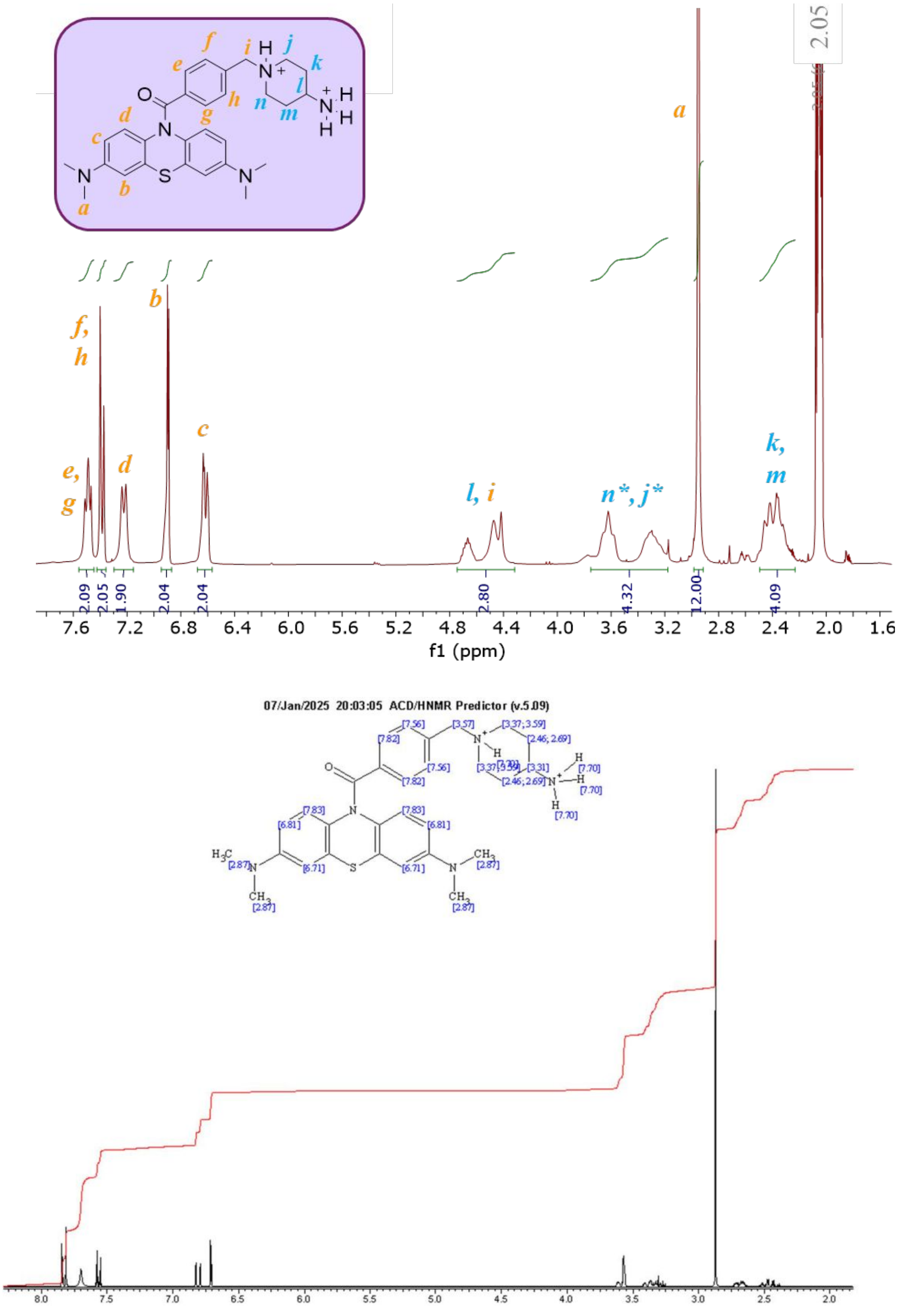

Figure S6. (Top) ^{1}H-NMR spectrum of diprotonated amino δ-BLMB in CD_3COCD_3 (300 MHz), obtained as the bis(trifluoroacetate) salt, and (bottom) calculated spectrum in $CDCl_3$ using ACD/HRMN Predictor (v. 5.09). *Tentative assignment.

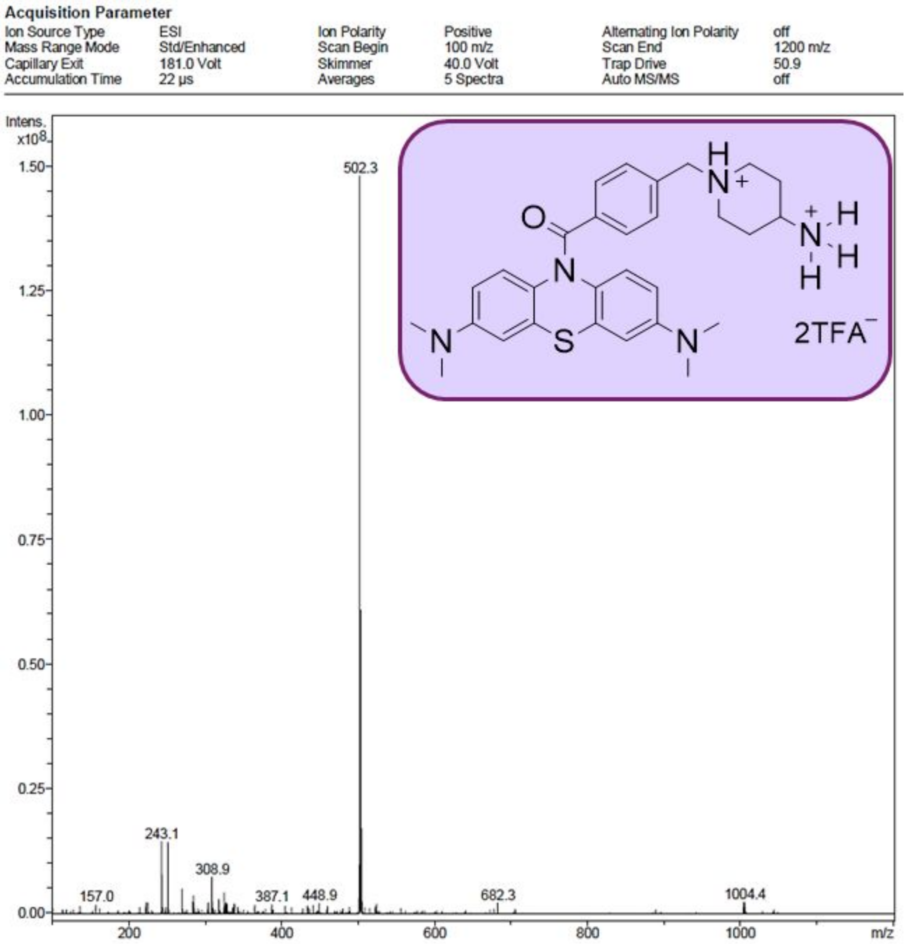

Figure S7. Electrospray ionization (positive ion detection mode) mass spectrum of aminated δ-BLMB, obtained in MeOH; m/z: calculated for [$C_{29}H_{35}N_5OS + H^+$]: 502.3, found: 502.3.

ii. Formation of the carboxymethoxyloxime of ZON

Zearalenone (ZON; 175 mg, 0.55 mmol) was dissolved in 2.5 mL of anhydrous MeOH and pyrrolidine (46 µL, 1.1 mmol) was added with stirring. Stirring was maintained for 5 min at room temperature. After this time, a second portion of pyrrolidine (46 µL, 1.1 mmol) was added and the mixture stirred for a further 5 min at room temperature. Then, *O*-(carboxymethyl)hydroxylamine hemichloride (60 mg, 0.55 mmol) was added. The mixture was then allowed to react at 55 °C for 15 min (Figure S8). Once the mixture cooled to room temperature, the solvent was removed under reduced pressure. Then, 5 mL of water were added, and the pH was adjusted to 3–4 by the dropwise addition of 1 mol L^{-1} HCl. The product precipitated as a white solid, which was collected by vacuum filtration. It was then washed three times with water and dried under vacuum (0.1 Torr)

for 24 h, resulting a yield of 93%. ^{1}H-NMR (700 MHz, CD_3OD): δ/ppm 6.99 (dd, J_1 = 15.2 Hz, J_2 = 1.8 Hz, 1H), 6.34 (d, J = 2.5 Hz, 1H), 6.19 (d, J = 2.5 Hz, 1H), 5.74 (ddd, J_1 = 15.2 Hz, J_2 = 9.8 Hz, J_3 = 4.3 Hz, 1H), 4.98 (m, 1H), 4.45 (m, 2.56H), 2.41–1.53 (m, 18H), 1.39–1.36 (m, 1H), 1.36 (d, J = 6.1 Hz, 3H). MS (ESI−) m/z: $[M - H]^+$ calculated for $[C_{20}H_{24}NO_7]^-$: 390.16; found: 390.0. The ^{1}H-NMR, ^{13}C/DEPT-135 NMR, and MS spectra are provided in Figures S9–S11, respectively.

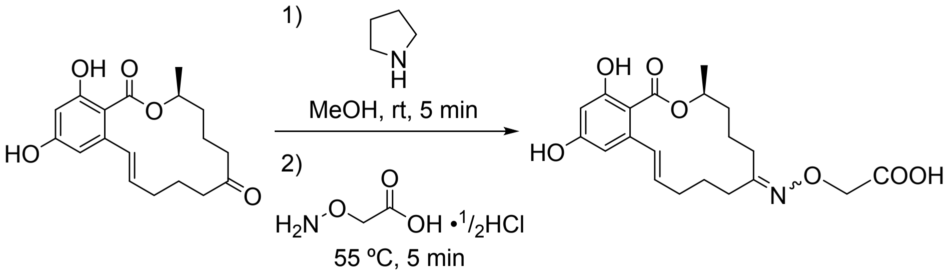

Figure S8. Synthetic route for the derivatization of the target mycotoxin endowed with a carboxyl group.

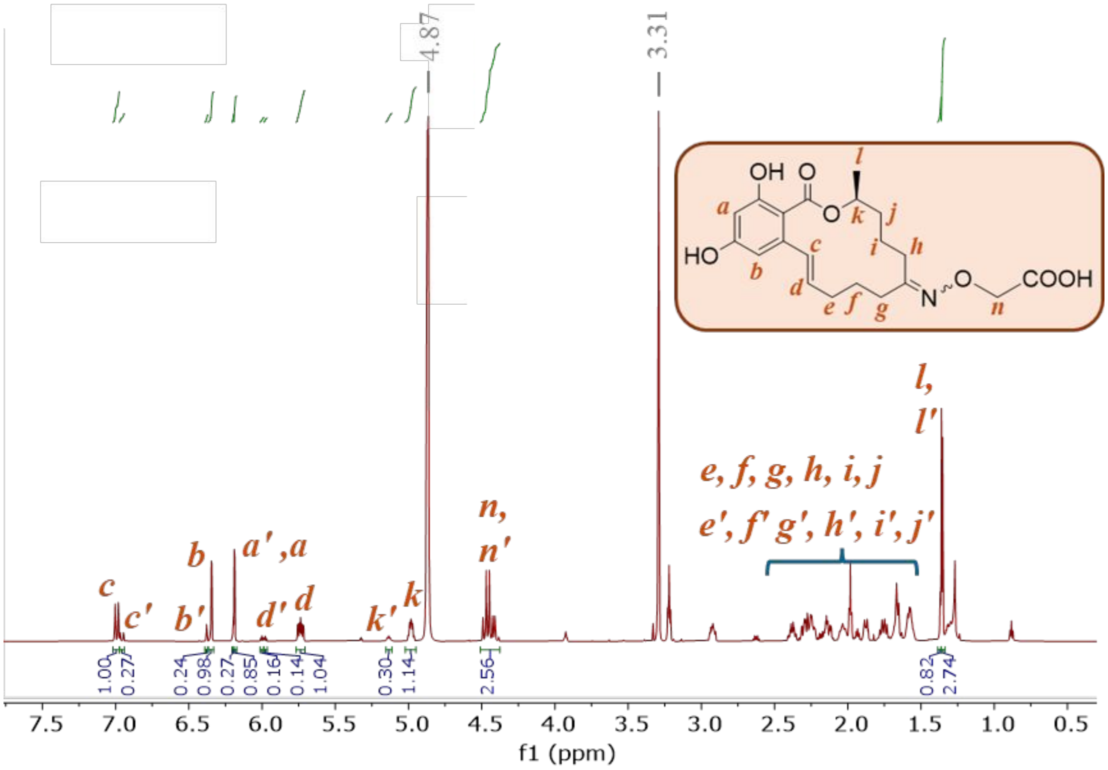

Figure S9. ^{1}H-NMR spectrum of the carboxymethoxyloxime of ZON in CD_3OD (700 MHz).

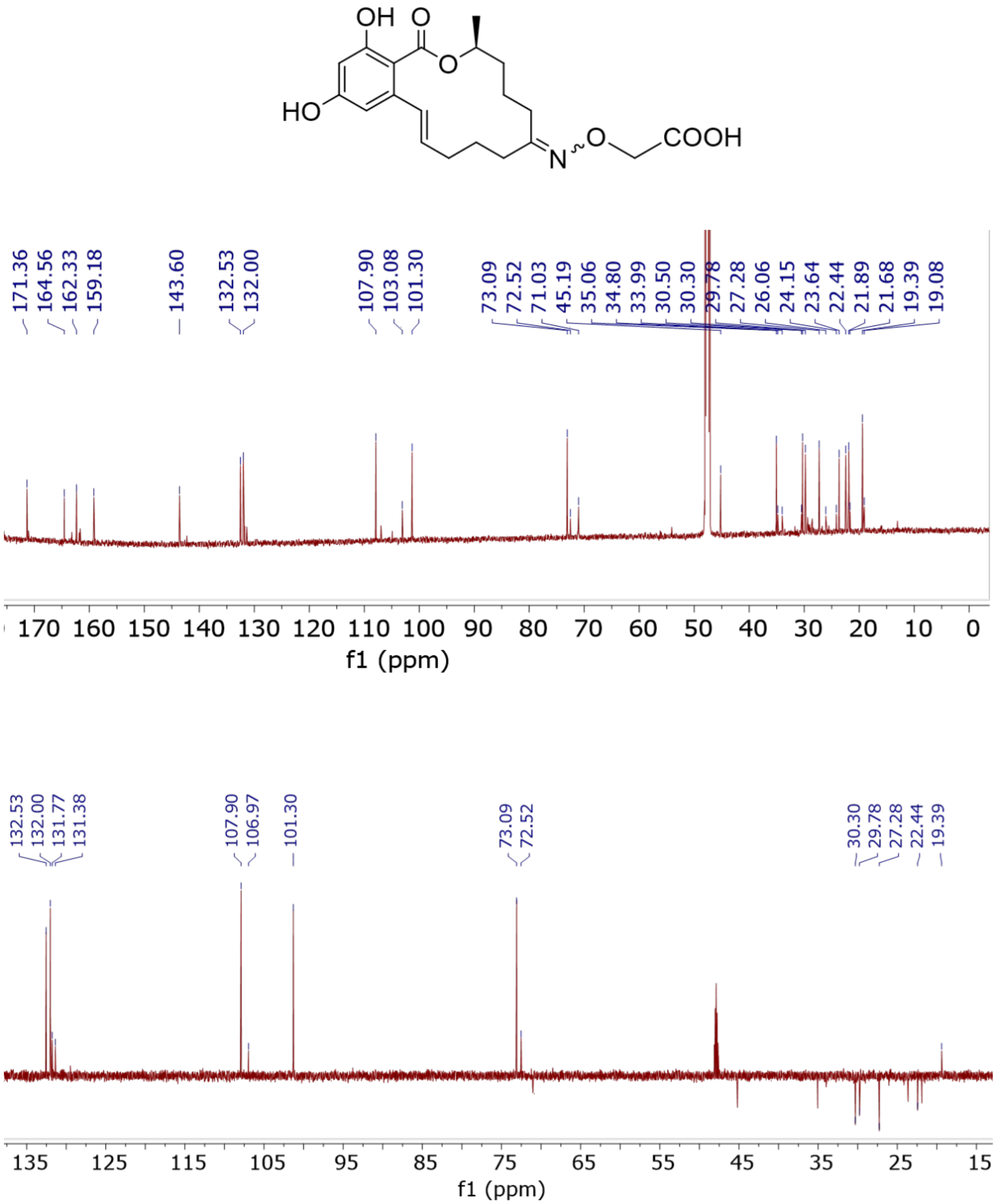

Figure S10. ^{13}C-NMR (top) and DEPT-135 (bottom) spectra of the carboxymethoxyloxime of ZON in CD_3OD (700 MHz).

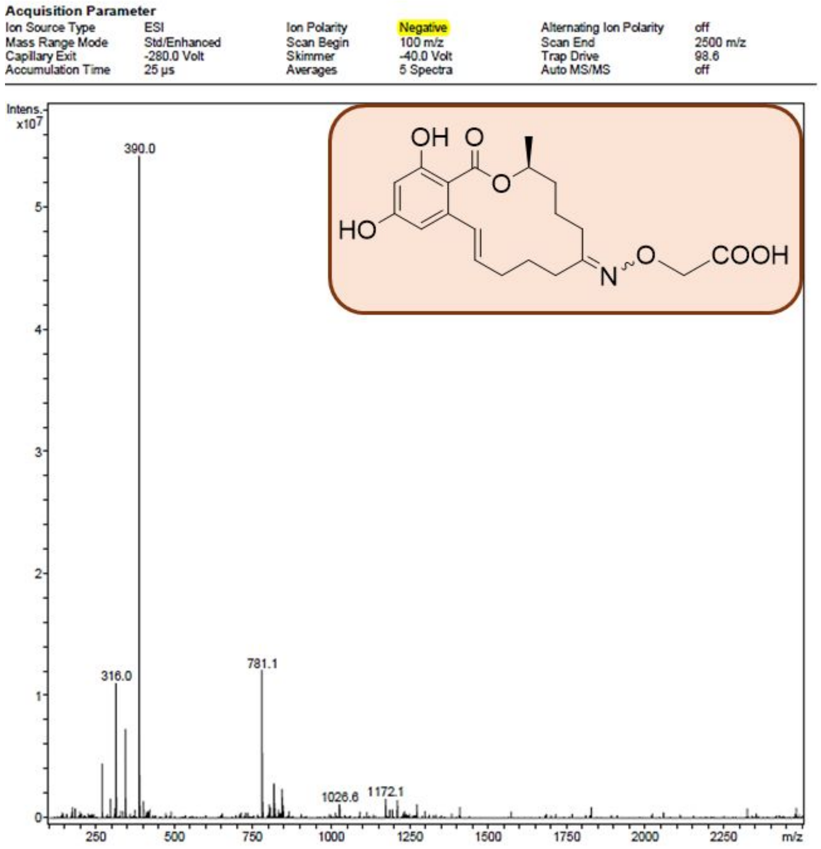

Figure S11. Electrospray ionization (negative ion detection mode) mass spectrum of the carboxymethyloxime of ZON, obtained in MeOH; m/z calculated for $[C_{20}H_{24}NO_7]^-$: 390.16, found: 390.0.

iii. Amide coupling

ZON carboxymethoxyloxime (23 mg, 60 µmol) was dissolved in 5 mL of anhydrous DMF. Then, *N,N*-diisopropylethylamine (52 µL, 300 µmol) was added to the mixture, which was allowed to react for 5 min. The mixture was then cooled to 0 °C, after which HATU (46 mg, 120 µmol) was added to activate the carboxylate group. Stirring was maintained for 1 h.

The aminated δ-BLMB bis(trifluoroacetate) salt (44 mg, 60 µmol) was subsequently added and the reaction was carried out under reflux for 2 h (Figure S12). To remove the DMF, a small amount of water was added, followed by four extractions with DCM. The resulting mixture was concentrated to dryness using a rotary evaporator. The crude reaction product was then purified by column chromatography (small-diameter, short column) using a 96:4 (v/v) dichloromethane–methanol mixture. The yield of the pure product was 50%. The R_f value was 0.32 on silica gel TLC

using a 96:4 (v/v) DCM–MeOH elution. The purified product was dried in a vacuum oven at room temperature (0.1 Torr). ^{1}H-NMR (300 MHz, $CDCl_3$): δ/ppm 7.62–7.46 (m, 3H), 7.45–7.36 (m, 3H), 6.79–6.72 (m, 2H), 6.57–6.24 (m, 6H), 5.91–5.63 (m, 1H), 4.47–4.39 (m, 2H), 3.44–3.29 (m, 3H), 2.92 (s, 14H), 2.79–2.75 (m, 2H), 2.74–2.60 (m, 3H), 2.50–1.47 (m, 32H). MS (ESI+) m/z: $[M]^+$ calculated for $[C_{49}H_{58}N_6O_7S + H^+]$: 875.41; found: 875.4. The ^{1}H-NMR and MS spectra are shown in Figure S13 and Figure S14, respectively.

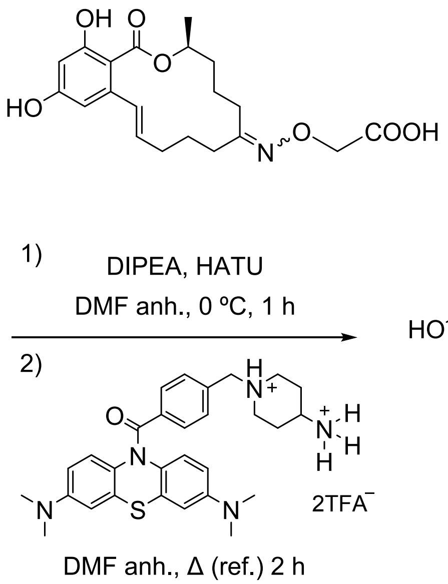

Figure S12. Synthetic route for the preparation of the BLMB-ZON conjugate from the derivatives shown in Figures S5 and S8.

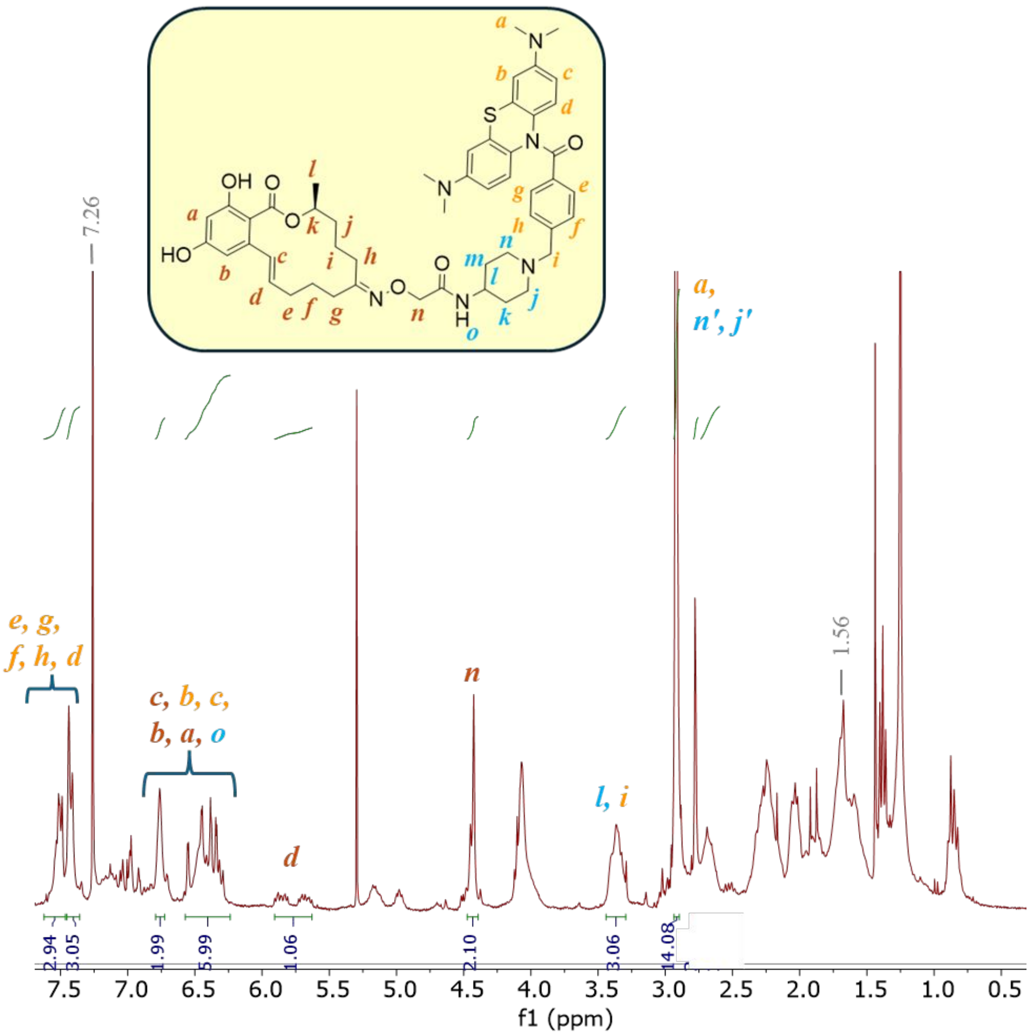

Figure S13. ^{1}H-NMR spectrum of the BLMB-ZON conjugate in $CDCl_3$ (300 MHz). Due to the spectral and molecular complexity, the indicated assignments are tentative.

Acquisition Parameter

Source Type	ESI	Ion Polarity	Positive	Set Nebulizer	3.0 Bar
Focus	Active	Set Capillary	4500 V	Set Dry Heater	200 °C
Scan Begin	50 m/z	Set End Plate Offset	-500 V	Set Dry Gas	9.0 l/min
Scan End	1300 m/z	Set Collision Cell RF	1000.0 Vpp	Set Divert Valve	Waste

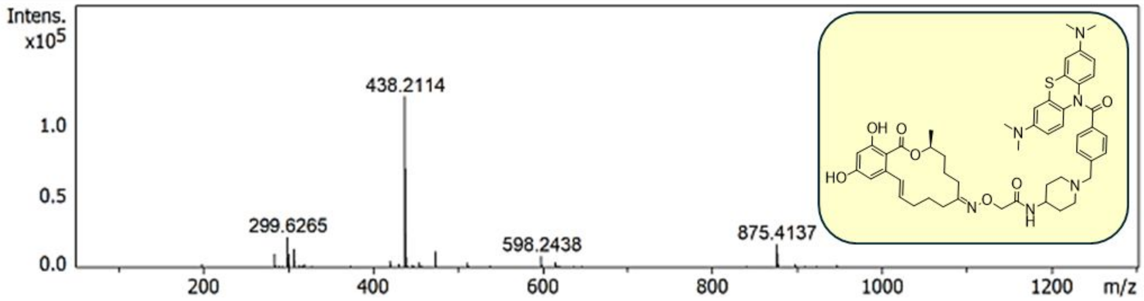

Figure S14. High-resolution electrospray ionization (positive ion detection mode) mass spectrum of the BLMB-ZON conjugate dissolved in EtOH; m/z calculated for $[C_{49}H_{58}N_6O_7S + 2H]^{2+}$: 438.2117, found: 438.2114.

Synthesis of BLMB-CO_2H

δ-BLMB (50 mg, 0.11 mmol) was dissolved in 10 mL of an ACN−water mixture (95:5, v/v), and 4-piperidinecarboxylic acid (74 mg, 0.57 mmol) was added to the solution in a 30 mL microwave reactor (Figure S15). The reaction mixture was maintained at 120 °C for 1.5 h under microwave irradiation with continuous stirring.

After cooling to room temperature, the interested product was extracted with DCM and water adjusted to pH 3–4 by dropwise addition of 1 mol L^{-1} HCl. The organic layer was separated and concentrated under reduced pressure. The R_f value was 0.44 on silica gel TLC previously treated with hexane-triethylamine (95:5, v/v), using chloroform–MeOH (9:1, v/v) as the eluent. TLC spots were visualized with ninhydrin solution spraying followed by heating. The purified product was dried in a vacuum oven at room temperature (0.1 Torr). Due to the complexity of the ^{1}H-NMR spectrum because of the slow rotation around the OC−N bond, even at high field, the MS (ESI+, exact mass) was recorded for the structural confirmation of BLMB-COOH; m/z: $[M]^+$ calculated for $[C_{30}H_{34}N_4O_3S + H]^+$: 531.2430; found: 531.2442 (Figure S16).

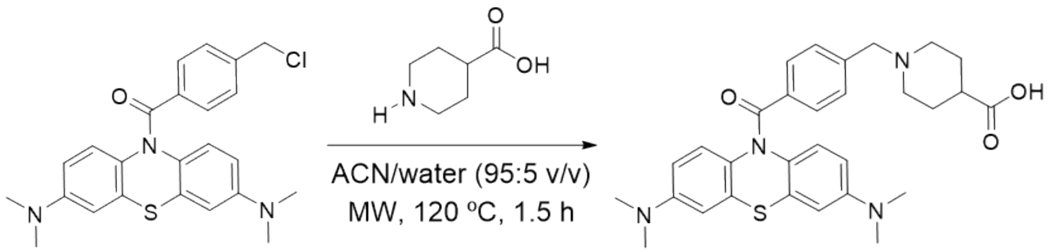

Figure S15. Synthetic route for the preparation of the BLMB-COOH conjugate from the chlorobenzyl derivative in Figure S2.

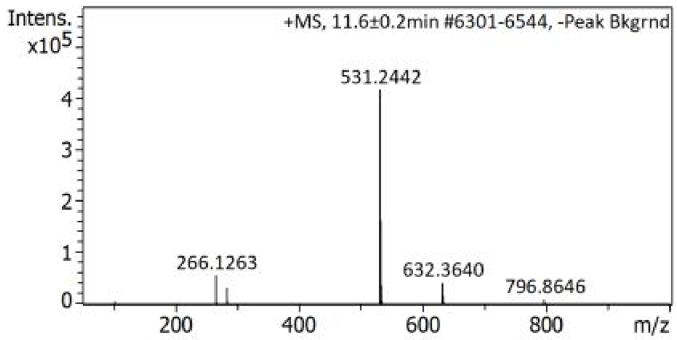

Figure S16. High-resolution electrospray ionization (positive ion detection mode) mass spectrum of the BLMB-COOH conjugate, obtained in MeOH; m/z calculated for $[C_{30}H_{34}N_4O_3S + H]^+$: 531.2430, found: 531.2442.

Synthesis of the BLMB–RAM conjugate

In an Eppendorf tube, 100 µL of 0.1 mol L^{-1} MES containing 0.9% NaCl was added and the pH adjusted to 4.7.[2] Then, 5 µL of a 2.69 × 10^{-3} M BLMB-CO_2H stock solution was incorporated. Under continuous stirring, 100 mmol L^{-1} of EDC (3.81 mg in 50 µL MES) was added dropwise, followed by 50 mmol L^{-1} sulfo-NHS (2.17 mg in 50 µL MES). The mixture was incubated for 15 min at room temperature with stirring.

After incubation, 100 µL of Ab-RAM in PBS (2.4 mg mL^{-1}, ~240 µg) was added, and the tube was placed in a rotating wheel for 2 h at room temperature. The mixture was then filtered and washed several times with 1X PBS using 100 kDa Amicon filters.

Conjugation of protein A/G–coated magnetic beads to Ab–ZON

Conjugation was performed directly on a 96-well plate. While the commercial protein A/G-coated magnetic microparticles (mBs) were being homogenized using a vortex mixer, several wells were blocked by the addition of 200 µL of a 3% bovine serum albumin (BSA) solution in purified water. Blocking was carried out at room temperature with shaking at 300 rpm for 1 h. Subsequently, the wells were washed three times with PBST.

The mBs were diluted from a 10 mg mL^{-1} stock solution to a concentration of 20 µg mL^{-1}, and the Ab#4 antibody was diluted from a 750 µg mL^{-1} stock solution to 5 µg mL^{-1}, using 100 mmol L^{-1} PBS as the diluent. In each well, 20 µL of the diluted mB suspension and 80 µL of the Ab#4 antibody solution were added. The mixture was then incubated at room temperature with shaking at 400 rpm for 30 min. Finally, the wells were washed three times with PBST, leaving the Ab#4–magnetic beads conjugate ready to use.

Protocols for studying interactions with BLMB–ZON conjugate

ELISA-like inhibition competitive immunoassay using immobilized OVA–ZON and Ab–ZON in the presence of the BLMB–ZON conjugate (Figure 23A)

A 96-well MaxiSorp plate was coated with 100 µL of OVA–ZON (50 ng mL^{-1}) in carbonate buffer (CB) at pH 9.6. The following day, the plate was washed three times with PBST and a first incubation was performed with 50 ng mL^{-1} each of antibodies Ab#1, Ab#2, Ab#3, and Ab#4, both

in the absence and in the presence of 100 nmol L^{-1} BLMB–ZON, to a final volume 100 µL of PBS. This incubation was carried out at room temperature with shaking at 400 rpm for 1 h. After incubation, the plate was washed again and incubated for a second time with 100 µL of a diluted HRP–RAM solution at 1:2000 in PBST. The plate was then washed and developed with 60 µL of 3,3′,5,5′-tetramethylbenzidine (TMB), followed by the addition of 60 µL of 2 mol L^{-1} H_2SO_4.

ELISA-like direct competitive assay with immobilized antibodies Ab#3 and Ab#4 and HRP–ZON in the presence of the BLMB–ZON conjugate (Figure S23B)

A MaxiSorp plate was coated with 60 µL of Ab#3 at 3.3 µg mL^{-1} in CB buffer at pH 9.6, and with 60 µL of Ab#4 under the same conditions. The following day, the plate was washed and blocked with 200 µL of 3% BSA, shaking at 300 rpm for 1 h. After blocking, the antibody-coated plate was washed three times with PBST and then incubated with 100 nmol L^{-1} HRP–ZON, both in the absence and presence of 1000 nmol L^{-1} BLMB–ZON conjugate, in a total volume of 100 µL. Incubation was carried out at room temperature with shaking at 400 rpm for 30 min. After incubation, the plate was washed and developed with 100 µL of TMB, followed by the addition of 100 µL of 2 mol L^{-1} H_2SO_4.

Among the four anti-zearalenone antibodies we produced and evaluated, Ab#4 was selected based on its highest sensitivity towards the BLMB-ZON conjugate in a **direct competitive** assay format. This format is the most representative of the photoamplified assay protocol (immobilized antibody).

Bead-based direct competitive assay using immobilized Ab#4 antibody and HRP–ZON in the presence of the BLMB–ZON conjugate (Figure S24)

A volume of 80 µL of a BLMB-ZON solution at different concentrations (0.001, 0.1, 1, 100, and 1000 nmol L^{-1}) was added to the wells containing magnetic beads previously conjugated with Ab#4. Samples were incubated under orbital shaking at 400 rpm at room temperature for 30 min. After the incubation, three washes with PBST were performed. Subsequently, 80 µL of an HRP–ZON solution at 100 nmol L^{-1} in PBS were incorporated and incubated at 300 rpm at room temperature for 30 min. Finally, the wells were washed three additional times with PBST and developed with 60 µL of TMB, followed by the addition of 60 µL of 2 mol L^{-1} H_2SO_4.

Bioassay protocol for photochemical amplification

Evaluation of the δ-BLMB and the BLMB–ZON conjugate in solution

In a first experiment, a solution containing 50 µmol L^{-1} δ-BLMB and 10 µmol L^{-1} tris(1,10-phenanthroline)ruthenium(II) dichloride (RP3) in ACN was prepared in a fluorescence cell (Hellma). This solution was illuminated with the 450-nm 50-mW diode laser for 1 min under magnetic stirring. The latter was followed by exposure to the 650-nm 150-mW diode laser for varying time intervals. After each interval, the emission spectrum between 665 and 700 nm was recorded (λ_{exc} = 650 nm). For the second experiment, a solution containing 50 µmol L^{-1} BLMB-ZON conjugate and 10 µmol L^{-1} RP3 was added to the cell. The interrogation and measurement process was carried out under the same conditions as in the previous experiment.

Bead-based direct competitive assay for ZON detection using BLMB–ZON

A volume of 80 µL of a 1000 nmol L^{-1} BLMB-ZON solution was added to each well of a clear 96-well plate containing magnetic beads previously conjugated with Ab#4 antibody, both in the presence and absence of 10000 nmol L^{-1} ZON. The samples were then incubated at 400 rpm and at room temperature for 30 min. After this period, the plate was washed three times with PBST. The samples were then dried in an oven at 37 °C for 30 min and reconstituted in 140 µL of ACN containing 10 µmol L^{-1} RP3. The sample wells were illuminated with the blue LED illuminator for 5 min while standing on an iced-cooled metal plate to prevent evaporation. The emission spectrum between 665 and 700 nm (λ_{exc} = 650 nm), corresponding to the emission band of the free MB, was collected. Then, 153 µmol L^{-1} δ-BLMB (10 µL of a 2.29 mmol L^{-1} stock solution) was added, after which the samples were illuminated with the red diode laser for 7 min. Finally, the characteristic MB fluorescence band was measured again.

Evaluation of the BLMB–RAM secondary antibody in the presence of Ab#3 and ZON (Figure S25)

To carry out the assay, 100 µL of 50 ng mL^{-1} OVA-ZON solution in carbonate buffer (pH 9.6) was added to each well of an untreated white microplate. To minimize leakage from the laser light exposure, alternate rows of the plate wells were used. Following a washing step, a first incubation was performed by adding 100 µL of a 1:1 (v/v) mixture of PBST and PBS containing 100 nmol

L^{-1} ZON and 100 ng mL^{-1} of the Ab#3 antibody. This incubation was carried out for 1 h at room temperature under orbital shaking at 400 rpm, followed by a second washing step.

Then, 100 µL of the BLMB-RAM secondary antibody solution diluted 1:500 in PBST was added, and the plate was incubated for 1 h at room temperature under orbital shaking at 400 rpm. After a third wash, the detection step was initiated by adding 140 µL of acetonitrile, followed by 1 µL of RP3 (final concentration: 10 µmol L^{-1}), after which the plate was illuminated with the blue LED illuminator for 5 min while standing on an ice-cooled surface.

Finally, 10 µL of δ-BLMB (final concentration 50 µmol L^{-1}) was added and the plate was illuminated again but this time with the red laser for 7 min while still standing on the cooled surface. The microplate reader was used to monitor the fluorescence of MB at 695 nm.

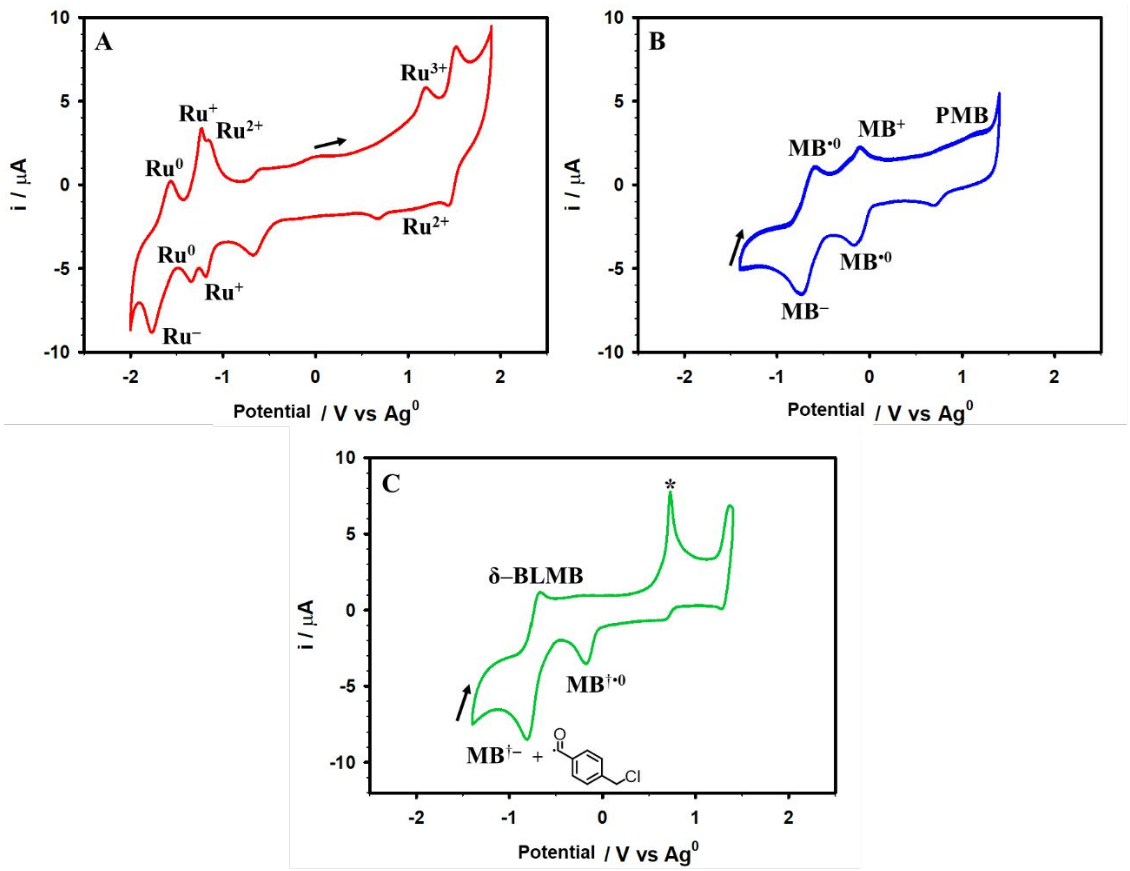

Figure S17. Cyclic voltammograms in ACN (containing 0.1 M $TBAPF_6$) under argon, obtained with a scan rate of 0.1 V s^{-1} (the arrows indicate the scan direction) for 0.1 mmol·L^{-1} solutions of: A) $[Ru(phen)_3]^{2+}$ ("Ru"), 2 Cl^-; B) MB; C) δ-BLMB. 'PMB' indicates the irreversible formation of a MB dimer. The asterisk (*) designates the appearance of a characteristic peak associated with adsorption of chemical species onto the electrode. The dagger symbol (†) means the different origin of otherwise identical redox species.

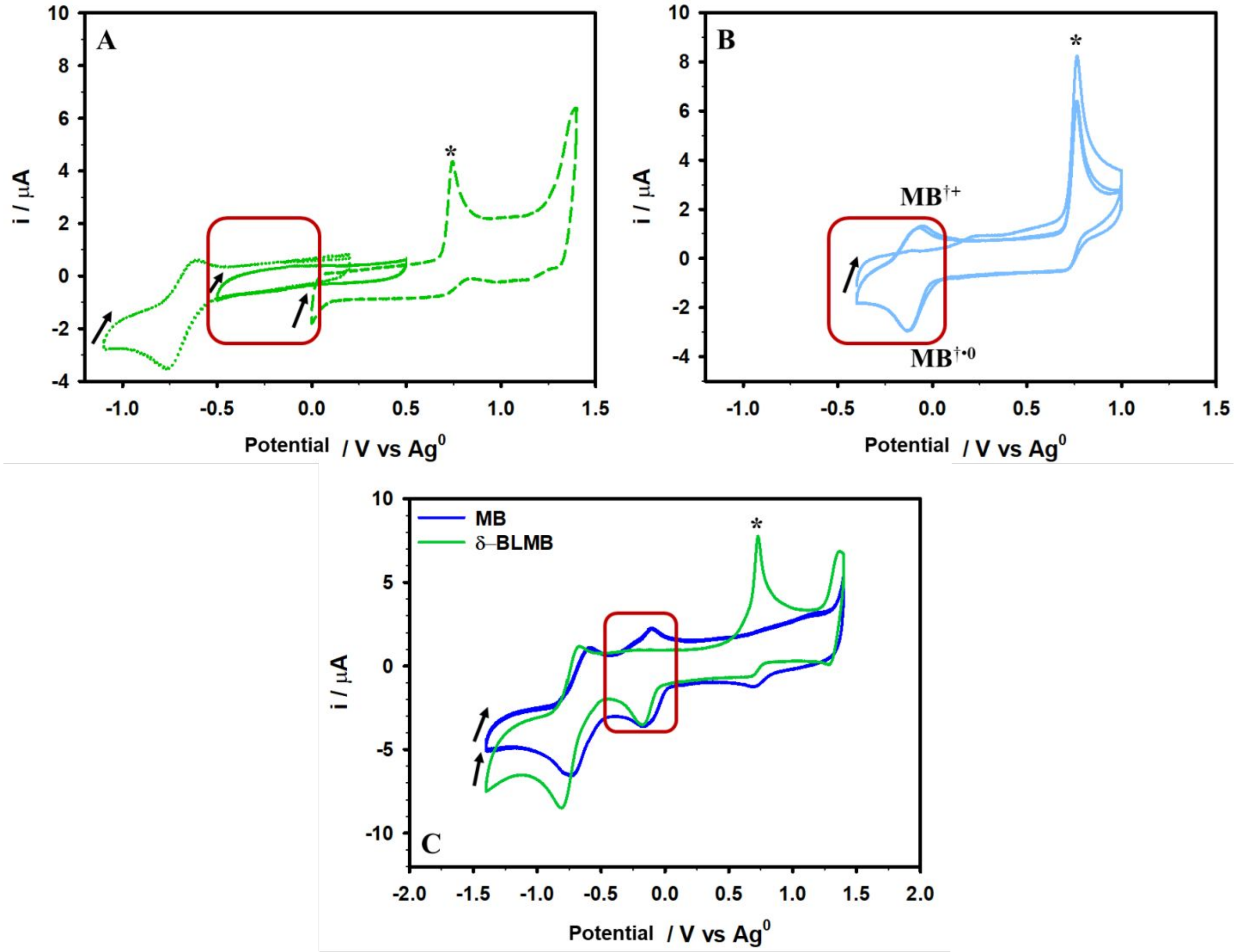

Figure S18. Cyclic voltammograms in ACN (containing 0.1 M $TBAPF_6$) under argon, measured on different potential windows at 0.1 V s^{-1} (the arrows indicate the scan direction). In A) and B), 0.1 mmol L^{-1} δ-BLMB solutions were investigated under different potential windows while, in C), the full voltammogram of δ-BLMB is compared to that of commercial MB. The red box highlights the region of interest, and the asterisk (*) indicates the appearance of a characteristic peak associated with adsorption of chemical species onto the electrode.

Table S1. Half-wave potentials (E) in ACN calculated from the anodic and cathodic peaks of voltammograms in Figure S17; the excited state potentials were calculated from the corresponding ground-state potentials (see text). The estimated uncertainty is ±0.02 V.[a,b]

E ($Ru^{3+/2+*}$) = −1.15 V	E ($MB^{\dagger+/\bullet 0}$) = −0.16 V
E (δ–BLMB/$MB^{\dagger-}$) = −0.75 V	E ($Ru^{2+/+}$) = −1.15 V
E ($Ru^{3+/2+}$) = 0.95 V	E ($MB^{+*/\bullet 0}$) = 1.64 V
E ($MB^{\dagger\bullet 0/-}$) = −0.67 V	E (Ar–$CO^{+/\bullet}$) = −0.82 V
E ($Ru^{2+*/+}$) = 0.95 V	

[a] The uncertainty of the excited state potentials does not account for the approximation $h\varpi_{0\text{-}0} \sim h\varpi_{max}$, being $h\varpi_{0\text{-}0}$ the energy of the 0-0 transition and $h\varpi_{max}$ the energy corresponding to the luminescence maximum. [b] "Ru" refers to [Ru(phen)$_3$].

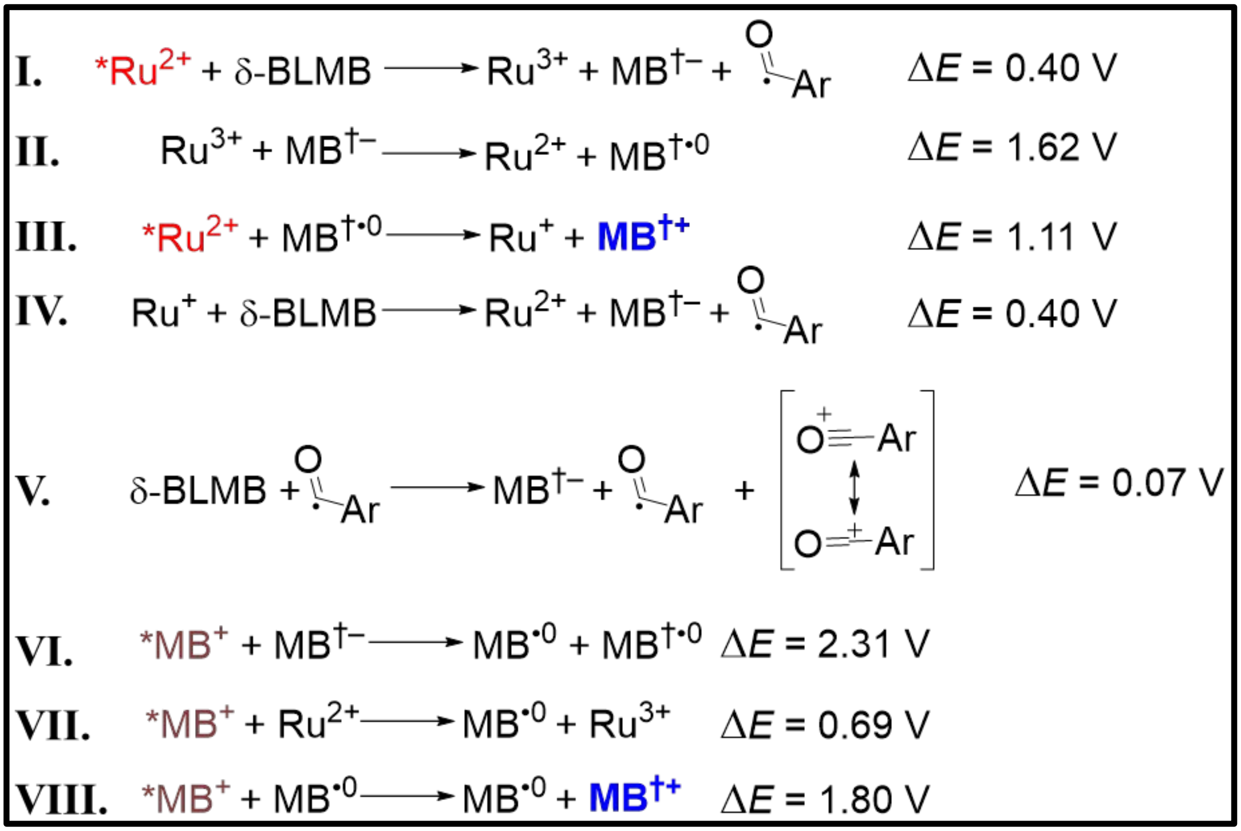

Figure S19. Suggested individual redox reactions involved in the photocatalytic mechanism, together with the ΔE values calculated from the corresponding half-wave potentials (**Table S1**). Species highlighted in red participate upon excitation at 450 nm; those in brown are activated with 650 nm light, and the one highlighted in blue corresponds to the species whose fluorescence is monitored as the analytical signal, confirming photoamplification via redox photocatalysis.

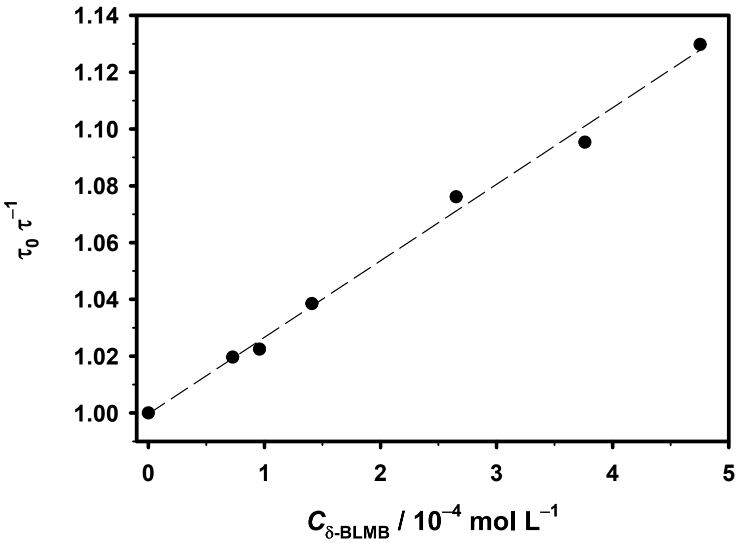

Figure S20. Fluorescence lifetime quenching plot ($\tau_0\tau^{-1} = 270 \times C_{\delta\text{-BLMB}} + 1$; $r^2 = 0.994$) of RP3 (2.94×10^{-5} mol L^{-1}) as a function of δ-BLMB concentration ($0 – 4.8 \times 10^{-4}$ mol L^{-1}) in ACN. The exponential RP3 emission decays were recorded at 605 nm ($\lambda_{exc} = 467$ nm).

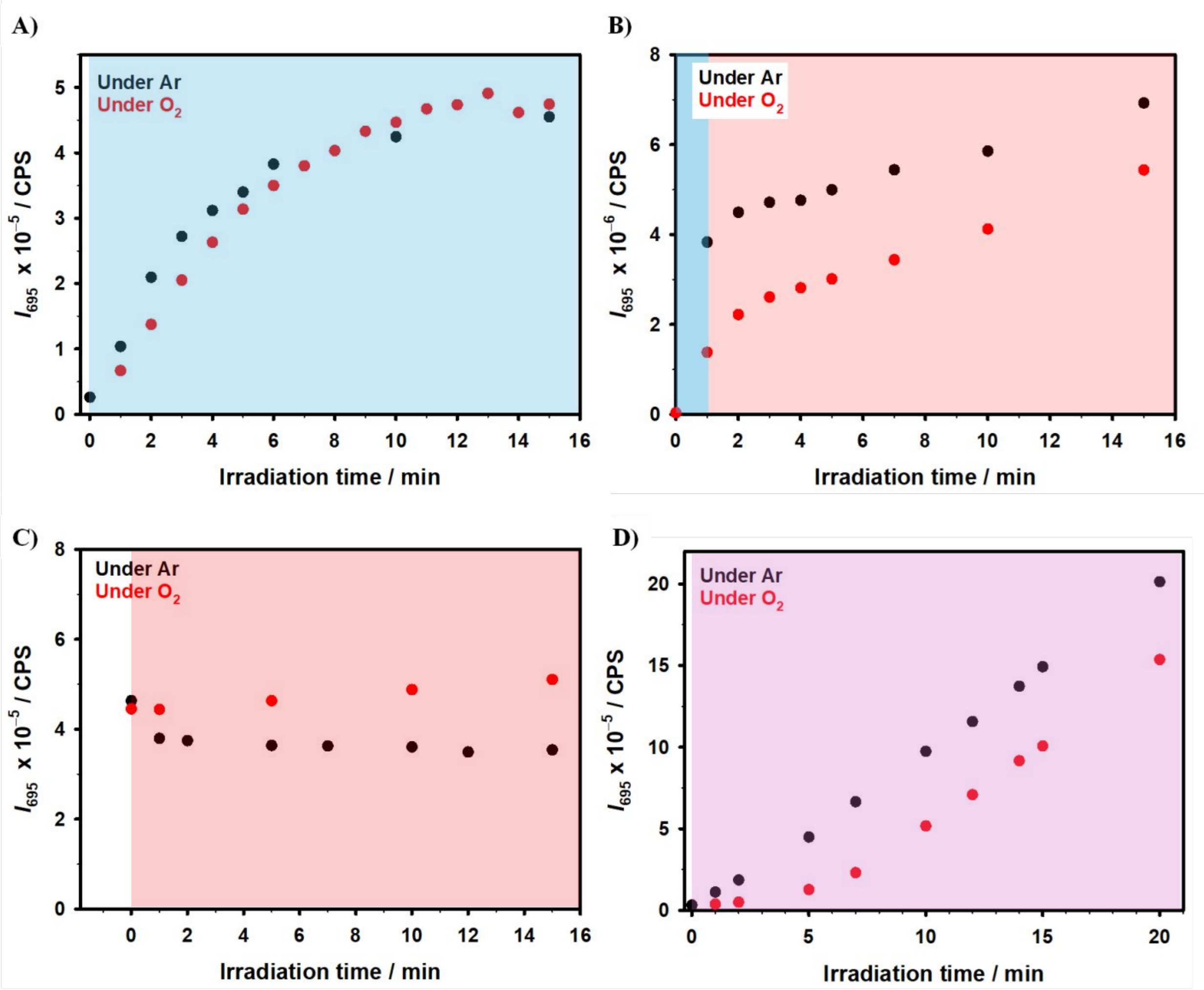

Figure S21. Fluorescence intensity at 695 nm (λ_{exc} = 650 nm) of δ-BLMB solutions (50 μmol L^{-1}) containing 10 μmol L^{-1} of the photocatalyst (PC) in ACN, in the presence (red points) and in the absence (black points) of O_2 (air). Excitation was performed according to the PC used. (A) RP3 as PC; 450 nm diode laser excitation; 2/2 nm emission monochromator slits. (B) RP3 as PC; 450 nm diode laser excitation for 1 min, followed by 650 nm diode laser illumination to excite MB^+ generated in the first step as PC; 5/5 nm emission slits. (C) MB as PC; 650 nm diode laser excitation; 2/2 nm emission slits. (D) PdTFPP as PC; 405 nm diode laser excitation; 5/5 nm emission slits. The colored zones indicate the different diode laser illumination.

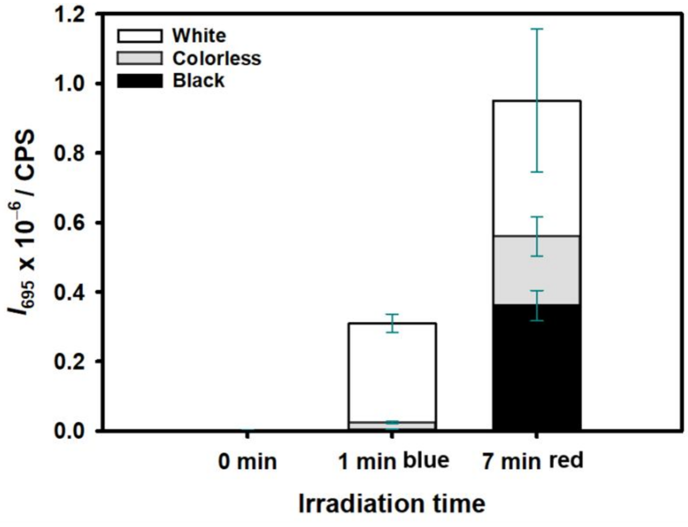

Figure S22. Fluorescence intensity at 695 nm (λ_{exc} = 650 nm) of the methylene blue formed in ACN after illuminating δ-BLMB (50 µmol L^{-1}) in the presence of RP3 (10 µmol L^{-1}) for 1 min with a blue-LED illuminator, followed by 7 min with a 650 nm diode laser, with the sample in black, clear, and white 96-well plates. Tests with white, black, and clear plates showed that white plates maximize the fluorescence signal due to enhanced reflection of the excitation and emission light, highlighting the impact of optical features on the observed amplification.

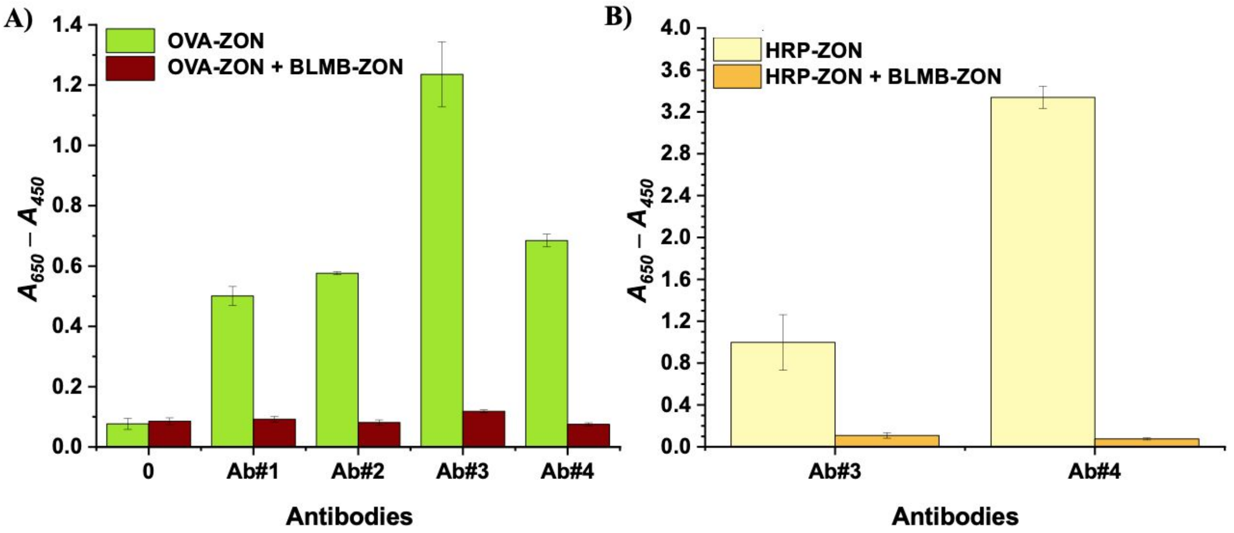

Figure S23. A) Indirect assay with an initial incubation of 50 ng mL^{-1} antibody with 50 ng mL^{-1} OVA-ZON, in the presence (burgundy bars) and in the absence (green bars) of 100 nmol L^{-1} (88 ng mL^{-1}) BLMB-ZON conjugate (in PBS), followed by a second incubation with HRP-RAM and development using TMB. B) Direct competitive assay using 3300 ng mL^{-1} antibody and 100 nmol L^{-1} HRP-ZON in the presence (orange bars) and in the absence (yellow bars) of 1000 nmol L^{-1} (875 ng mL^{-1}) BLMB-ZON conjugate (in PBS), with TMB development.

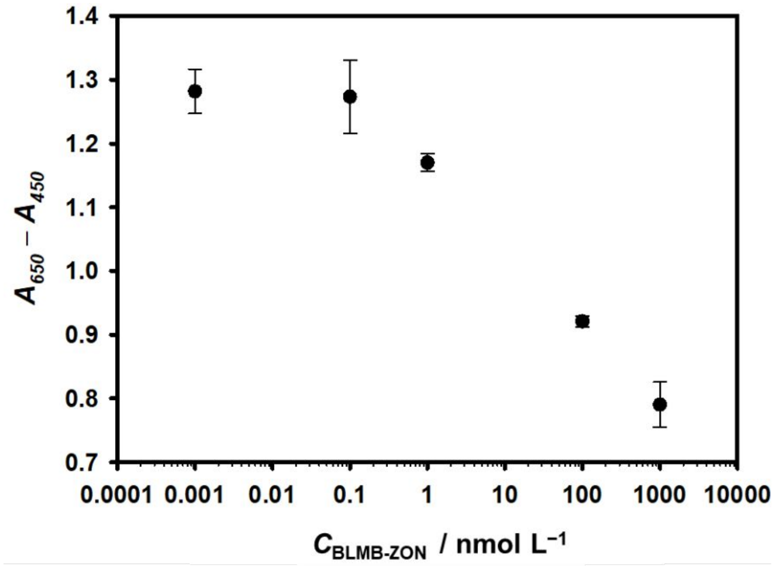

Figure S24. Direct competitive assay using magnetic microbeads functionalized with Ab#4 antibody and 100 nmol L^{-1} HRP-ZON, in the presence of varying concentrations of the BLMB-ZON conjugate (in PBS), after development with TMB.

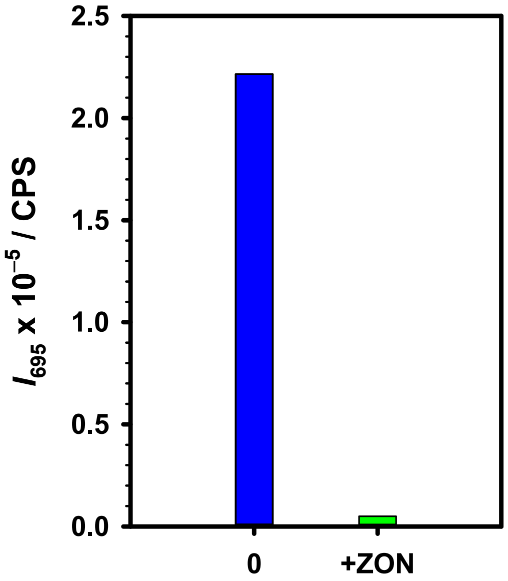

Figure S25. Fluorescence of MB at 695 nm using 100 ng mL^{-1} Ab#3 antibody and BLMB-RAM secondary antibody diluted 1:500, in the presence of 100 nmol L^{-1} ZON ('+ZON') and in the absence ('0') of ZON; the signal was recorded after 5 min of blue-LED irradiation and subsequent addition of 153 μmol L^{-1} δ-BLMB (pro-photocatalyst) with red-diode laser illumination for 7 min.

References

(1) Abad-Fuentes, A.; Agulló, C.; López-Puertollano, D.; Navarro-Fuertes, I.; Abad-Somovilla, A.; Mercader, J. V. Alternative Hapten Design for Zearalenone Immunoreagent Generation. *Toxins* **2022**, *14* (3), 185; https://doi.org/10.3390/toxins14030185.

(2) Thermo Fisher Scientific. EDC (1-ethyl-3-[3-dimethylaminopropyl]carbodiimide hydrochloride), User Guide. https://documents.thermofisher.com/TFS-Assets/LSG/manuals/MAN0017125_EDC_UG.pdf (accessed 2026-01-27).

(3) Wild, D., Editor. The Immunoassay Handbook: Theory and applications of ligand binding, ELISA and related techniques. 4th ed. Amsterdam: Elsevier; 2013.
